# A Greener and Efficient Method for Nucleophilic Aromatic Substitution of Nitrogen-Containing Fused Heterocycles

**DOI:** 10.3390/molecules23030684

**Published:** 2018-03-18

**Authors:** Joana F. Campos, Mohammed Loubidi, Marie-Christine Scherrmann, Sabine Berteina-Raboin

**Affiliations:** 1 Institute of Organic and Analytical Chemistry (ICOA), University of Orleans, UMR-CNRS 7311, BP 6759 F-45067 Orleans CEDEX 2, France; joana-filomena.mimoso-silva-de-campos@univ-orleans.fr (J.F.C.); m.loubidi@gmail.com (M.L.); 2 Institute of Molecular Chemistry and Materials of Orsay, UMR CNRS 8182, University of Paris-Sud, Building 420, 91400 Orsay, France; marie-christine.scherrmann@u-psud.fr

**Keywords:** PEG-400, nucleophilic aromatic substitution, nitrogen fused heterocycles

## Abstract

A simple and efficient methodology for the nucleophilic aromatic substitution of nitrogen-containing fused heterocycles with interesting biological activities has been developed in an environmentally sound manner using polyethylene glycol (PEG-400) as the solvent, leading to the expected compounds in excellent yields in only five minutes.

## 1. Introduction

Environmentally sustainable practices are increasingly being taken into consideration in medicinal chemistry and applied as far as possible by the various pharmaceutical companies and laboratories [1,2,3]. It is therefore necessary to provide chemists with effective methods for the development of complex structures under mild and green conditions. Green chemistry refers to the design of a process that minimizes the use and generation of hazardous substances [4]. As pointed out in [5,6,7], the solvent often represents the major part of the mass used in a reaction or a process, and chemists are therefore encouraged to use greener alternatives [8,9,10,11]. In this context, polyethylene glycols (PEGs), compounds with widespread industrial and medical applications [12,13], have attracted special attention as green solvents in various chemical transformations [14,15,16]. These rather inexpensive polymers are available in a wide range of molecular weights and are mainly produced from ethylene glycol, a by-product of the petrochemical industry, but can also be obtained from agricultural waste [17]. PEG400 is a viscous sustainable liquid soluble in water and many organic solvents. It has the advantage of being readily biodegradable as well as non-toxic, odourless, neutral, non-volatile, and non-irritating, which explains its use in a variety of pharmaceuticals and medications [12,13,18].

**Figure 1:**
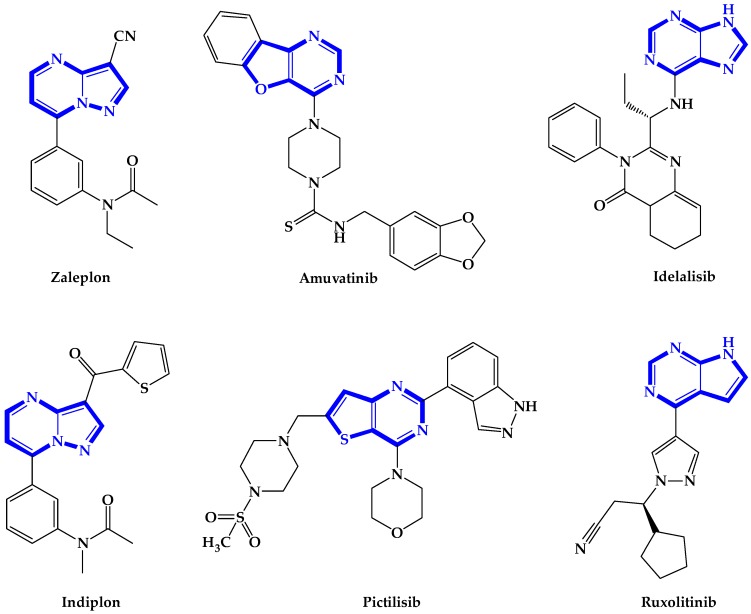
Structures of some Food and Drug Administration (FDA) approved drugs with the bicyclic scaffold reported in this article.

Substituted pyrimidine and pyrazine derivatives are a significant class of nitrogen-fused heterocycles, which are ubiquitous in many natural products and biologically active compounds in agrochemistry as well as in the pharmaceutical area. Over the past few decades, more and more drugs with fused bicyclic pyrimidine and pyrazine scaffolds have been approved by the Food and Drug Administration (FDA) for their significant biological activities, such as antitumor activities [19,20,21] and insomnia disorder [22]. In the major cases, the fused bicyclic pyrimidines exhibit an anticancer function by targeting different kinases [20,21], such as epidermal growth factor receptor (EGFR), Bruton’s tyrosine kinase (BTK), Janus kinase (JAK), and phosphatidylinositol 3 kinase (PI3K), (Figure 1).

These compounds play an important role in drug discovery and development [23,24]. In view of our interest in the development of green chemistry procedures [25,26,27,28,29,30,31,32,33,34], we report herein the use of PEG400 as an efficient medium for nucleophilic aromatic substitution (S_N_Ar) of some nitrogen-containing fused heterocycles with various amines. S_N_Ar involving amines have been carried out and studied in conventional organic solvents [35,36,37,38,39] but also in non-volatile alternative media such as ionic liquids [40,41,42,43,44], but, to the best of our knowledge, this reaction has never been reported using PEG as the solvent.

## 2. Results and Discussion

For this study the chloro compounds were chosen as starting materials. In all cases the reactions were conducted without additional base and the results obtained are given by class of heterocycles. In the first attempts, we explored the temperature parameter, however attempts performed below 120 °C did not give the desired product and this was consistent for all the scaffolds chosen as the starting material. This is likely due to the poor solubility problem of the reagents at temperatures below 120 °C.

### 2.1. From 4-Chloro-2-methylimidazo[1,5-a]pyrimidine-8-carbonitrile

Commercially available 4-chloro-2-methylimidazo[1,5-*a*]pyrimidine-8-carbonitrile was reacted with various primary or secondary amines in PEG 400 as the solvent without additional base, initially at room temperature. However, these conditions were not appropriate due to the lack of solubility of the mixture of starting materials. At 120 °C, all the reactants were soluble and we were pleased to observed the formation of 2-methylimidazo[1,5-*a*]pyrimidine-8-carbonitrile with an amino group in position 4 within only 5 min.

**Scheme 1:**
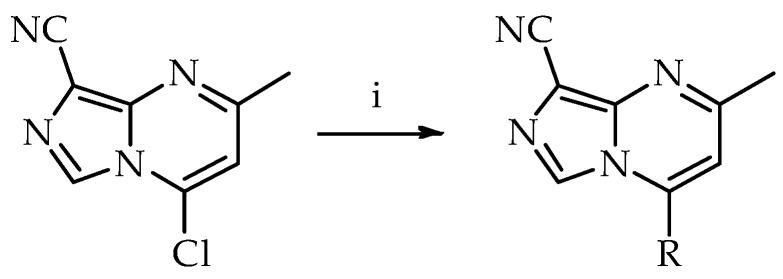
Synthesis of **1**–**6**. *Reagents and conditions*: (i) amino derivative (2 equiv.), PEG400, 120 °C, 5 min, 70–95%.

Compounds **2** to **6** were obtained with good yields (81 to 95%). The lowest yield (70%, entry 6, compound **1**) is due to the strong electro-withdrawing effect of the trifluoromethyl group in the ortho position of the aniline, which is an amine that is already less nucleophilic than aliphatic amines (Scheme 1, Table 1). PEG-400 is a very effective solvent to generate these amino substituted heterobicyclic compounds which can be used as fungicides [45].

These reaction conditions with amine derivative (2 equiv.) in PEG 400 at 120 °C for 5 min were applied to other nitrogen-containing fused heterocycles.

### 2.2. From 7-Chloro-5-methyl-[1,2,4]triazolo[1,5-a]pyrimidine

[1,2,4]Triazolo[1,5-*a*]pyrimidines are a highly interesting class of fused heterocycles due to their valuable biological properties. Some [1,2,4]-triazolo[1,5-*a*]pyrimidines possess herbicidal activity [46,47], while others can act as antifungal [48,49], antitubercular [50,51] and antibacterial [52] agents. Polycyclic systems containing a [1,2,4]triazolo[1,5-*a*]-pyrimidine moiety are reported as antitumor agents [53,54], as corticotropin releasing factor 1 receptor antagonists [55] or calcium channel modulators [56] and they can also be used for the treatment of Alzheimer’s disease [57] and insomnia [58].

**Scheme 2:**
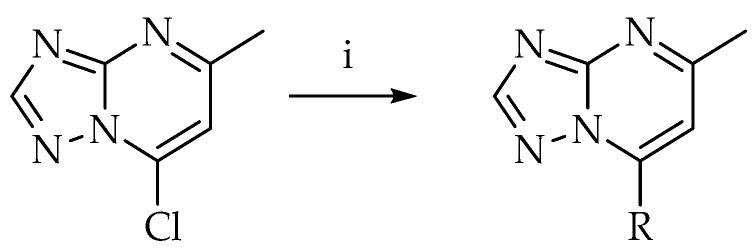
Synthesis of **7**–**10**. *Reagents and conditions*: (i) amino derivative (2 equiv.), PEG400, 120 °C, 5 min, 79–89%.

The commercially available 7-chloro-5-methyl-[1,2,4]triazolo[1,5-*a*]pyrimidine was submitted to the same conditions as 4-chloro-2-methylimidazo[1,5-*a*]pyrimidine-8-carbonitrile in PEG-400 as solvent at 120 °C without additional base. In this case also we were able to synthesize the desired compounds 7 to 10 in only 5 min in good yields (Scheme 2, Table 2).

### 2.3. From 8-Chloro-[1,2,4]triazolo[4,3-a]pyrazine

**Scheme 3:**
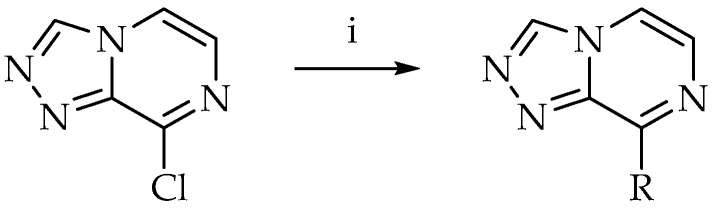
Synthesis of **11**–**14**. *Reagents and conditions*: (i) amino derivative (2 equiv.), PEG400, 120 °C, 5 min, 73–99%.

The fused triazole-moiety can be found in a variety of biologically active compounds including antibacterial [59], anti-inflammatory [60,61], antimicrobial [62], antiplatelet [63], anticonvulsant and antidiabetic [64] agents. In particular, bicyclic fused 1,2,4-triazole derivatives are an important group of heterocycles and have been the subject of studies from various academic and industrial groups in the recent past due to their biological versatility [65]. The commercially available 8-chloro-[1,2,4]triazolo[4,3-*a*]pyrazine underwent the analogue nucleophilic aromatic substitution in the same conditions with various amines, leading to the expected compounds in good yields (73% to 99%). The reactions were rapid as for the previous examples (Scheme 3, Table 3).

### 2.4. From 4-Chlorofuro and thieno[3,2-d]pyrimidine

**Scheme 4:**
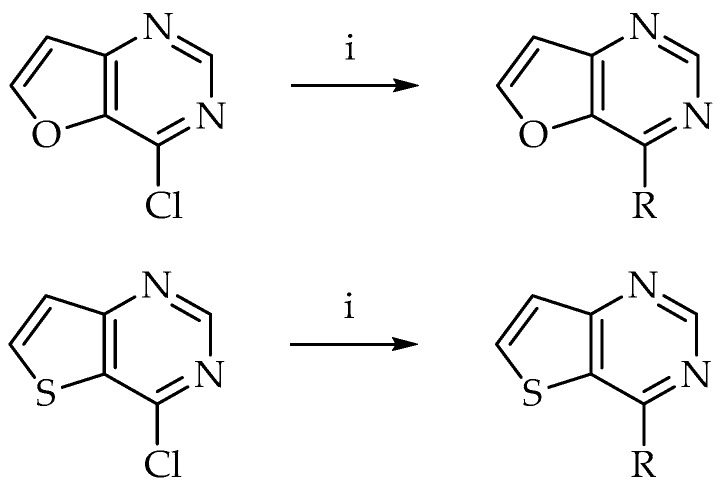
Synthesis of **15**–**24**. *Reagents and conditions*: (i) amino derivative (2 equiv.), PEG400, 120 °C, 5 min, 71–99%.

Thienopyrimidines are fused heterocyclic ring systems; structurally they resemble purines, and they have considerable pharmacological potential. They are known to play a crucial role in various disease conditions. Thieno[2,3-*d*]pyrimidine derivatives have been explored for their inhibitory activities towards various protein kinase enzymes [66]. Furopyrimidine heterocyclic ring systems are structural analogues of purines which have been subjected to biological investigations to assess their potential therapeutic usefulness [67]. Furopyrimidines have attracted considerable attention because of their great practical potential as antiviral [68,69,70], antimicrobial [71] and antitumor agents [72,73]. Starting from commercially available 4-chlorofuro and thieno[3,2-*d*]pyrimidine we obtained the same results, good to excellent yields (71% to 99%), for the desired compounds **15** to **24** under the same conditions (Scheme 4, Table 4).

## 3. Materials and Methods

### 3.1. General Methods

All reagents were purchased from commercial suppliers and were used without further purification. THF was dried with a GT S100 drying station immediately prior to use. The reactions were monitored by thin-layer chromatography (TLC) analysis using silica gel (60 F254) plates. Compounds were visualized by UV irradiation. Flash column chromatography was performed on silica gel 60 (230–400 mesh, 0.040–0.063 mm). Melting points (mp (°C)) were taken on samples in open capillary tubes and are uncorrected. The infrared spectra of compounds were recorded on a Nicolet iS10 spectrophootometer (Thermo Scientific, Villebon-sur-Yvette, France). ^1^H- and ^13^C-NMR spectra were recorded on an Avance II spectrometer at 250 MHz (^13^C, 62.9 MHz) and on an Avance III HD nanobay 400 MHz (^13^C 100.62 MHz) (Bruker, Wissembourg, France). Chemical shifts are given in parts per million from tetramethylsilane (TMS) or deuterated solvent (MeOH-*d*_4_, Chloroform-*d*) as internal standard. The following abbreviations were used for the proton spectra multiplicities: b: broad, s: singlet, d: doublet, t: triplet, q: quartet, p: pentuplet, m: multiplet. Coupling constants (*J*) are reported in Hertz (Hz). High-resolution mass spectra (HRMS (ESI)) were performed on a Maxis Bruker 4G by the “Federation de Recherche” ICOA/CBM (FR2708) pFlatform.

### 3.2. General Procedure for the Synthesis of ***1*** to ***24***

A mixture of chloro compound (50 mg) and amine derivative (2 equiv.) in PEG 400 (2 mL) was stirred at 120 °C for 5 min. After completion the reaction was then cooled to room temperature. DCM and water were added and the phases were separated. The aqueous phase was extracted with DCM and the organic phase was dried and filtered. The removal of solvent gave the product as a white solid.

*2-Methyl-4-(piperidin-1-yl)imidazo[1,5-a]pyrimidine-8-carbonitrile* (**1**) [74]. From 4-chloro-2-methyl-imidazo[1,5-*a*]pyrimidine-8-carbonitrile (50 mg; 0.260 mmol) and piperidine (44 mg; 0.520 mmol), (54 mg, 87%), m.p 164–166 °C. ^1^H-NMR (250 MHz, CDCl_3_) δ 1.74–1.81 (m, 6H), 2.51 (s, 3H), 3.25–3.29 (m, 4H), 6.04 (s, 1H), 7.80 (s, 1H) ppm. ^13^C-NMR (63 MHz, CDCl_3_) δ 24.0 (2xCH), 25.2 (2xCH), 50.4 (2xCH), 96.5 (CH), 100.8 (C), 115.1 (C), 123.8 (CH), 145.9 (C), 149.5 (C), 162.7 (C) ppm.

*2-Methyl-4-morpholinoimidazo[1,5-a]pyrimidine-8-carbonitrile* (**2**). From 4-chloro-2-methylimidazo[1,5-*a*]pyrimidine-8-carbonitrile (50 mg; 0.260 mmol) and morpholine ( 45 mg; 0.520 mmol), (58 mg, 92%), m.p 178–180 °C. ^1^H-NMR (250 MHz, CDCl_3_) δ 2.60 (s, 3H), 3.29–3.32 (m, 4H), 3.94–3.98 (m, 4H), 6.11 (s, 1H), 7.88 (s, 1H) ppm. ^13^C-NMR (63 MHz, CDCl_3_) δ 25.2 (CH), 49.5 (2xCH), 66.0 (2xCH), 96.9 (CH), 100.0 (C), 114.7 (C), 123.4 (CH), 145.6 (C), 148.8 (C), 162.6 (C) ppm. HRMS: calcd for C_12_H_14_N_5_O [M + H]^+^ 244.1193, found 244.1192.

*4-((2R,6S)-2,6-Dimethylmorpholino)-2-methylimidazo[1,5-a]pyrimidine-8-carbonitrile* (**3**). From 4-chloro-2-methylimidazo[1,5-*a*]pyrimidine-8-carbonitrile (50 mg; 0.260 mmol) and *cis*-2,6-dimethyl-morpholine (60 mg; 0.520 mmol), (67 mg, 95%), m.p 252–254 °C. ^1^H-NMR (400 MHz, CDCl_3_) δ 1.27 (d, *J* = 6.3 Hz, 6H), 2.56 (s, 3H), 2.68–2.73 (m, 2H), 3.44 (d, *J* = 11.8 Hz, 2H), 3.91–3.95 (m, 2H), 6.08 (s, 1H),7.84 (s, 1H) ppm. ^13^C-NMR (100.6 MHz, CDCl_3_) δ18.7 (2xCH), 25.2 (CH), 54.5 (2xCH), 71.1 (2xCH), 96.9 (CH), 101.4 (C), 114.8 (C), 123.5 (CH), 145.7 (C), 148.4 (C), 162.7 (C) ppm. HRMS: calcd for C_14_H_18_N_5_O [M + H]^+^ 272.1506, found 272.1503.

*4-(Dibutylamino)-2-methylimidazo[1,5-a]pyrimidine-8-carbonitrile* (**4**) [74]. From 4-chloro-2-methyl-imidazo[1,5-*a*]pyrimidine-8-carbonitrile (50 mg; 0.260 mmol) and di-*n*-butylamine (67 mg; 0.520 mmol), (60 mg, 81%), m.p 145–147 °C. ^1^H-NMR (250 MHz, CDCl_3_) δ 0.9 (t, *J* = 7.3 Hz, 6H),1.22–1.38 (m, 4H), 1.53–1.65 (m, 4H), 2.51 (s, 3H), 3.29–3.35 (m, 4H), 6.01 (s, 1H), 7.85 (s, 1H) ppm. ^13^C-NMR (63 MHz, CDCl_3_) δ 13.7 (2xCH), 20.1 (2xCH), 25.1 (CH), 29.0 (2xCH), 50.2 (2xCH), 96.9 (CH), 100.6 (C), 115.2 (C),123.9 (CH), 146.6 (C), 148.2 (C), 162.3 (C) ppm.

*4-(((3s,5s,7s)-Adamantan-1-yl)amino)-2-methylimidazo[1,5-a]pyrimidine-8-carbonitrile* (**5**). From 4-chloro-2-methylimidazo[1,5-*a*]pyrimidine-8-carbonitrile (50 mg; 0.260 mmol) and adamantylamine (78 mg; 0.520 mmol), (68 mg, 85%), m.p 293–295 °C. ^1^H-NMR (400 MHz, CDCl_3_) δ 1.74–1.81 (m, 6H), 2.13 (s, 6H), 2.25 (s, 3H), 2.54 (s, 3H), 4.88 (s, 1H), 6.03 (s, 1H), 7.90 (s, 1H) ppm. ^13^C-NMR (100.6 MHz, CDCl_3_) δ 25.6 (CH), 29.4 (3xCH), 35.9 (2xCH), 41.8 (3xCH), 54.07 (C), 80.0 (CH), 90.3 (CH), 100.2 (C), 115.3 (C), 120.2 (CH), 142.12 (C), 145.9 (C), 161.93 (C) ppm. HRMS: calcd for C_18_H_22_N_5_ [M + H]^+^ 308.1869, found 308.1870.

*2-Methyl-4-((2-(trifluoromethyl)phenyl)amino)imidazo[1,5-a]pyrimidine-8-carbonitrile* (**6**). From 4-chloro-2-methylimidazo[1,5-*a*]pyrimidine-8-carbonitrile (50 mg; 0.260 mmol) and 2-trifluoromethylaniline (83 mg; 0.520 mmol), (57 mg, 70%), m.p 205–207 °C. ^1^H-NMR (400 MHz, CDCl_3_) δ 2.23 (s, 3H), 5.25 (s, 1H), 6.94 (d, *J* = 7.9 Hz, 1H), 7.20 (t, *J* = 7.6 Hz, 1H), 7.52 (t, *J* = 7.6 Hz, 1H), 7.68 (d, *J* = 7.8 Hz, 1H), 8.25 (s, 1H), 10.30 (s, 1H) ppm.^13^C-NMR (100.6 MHz, CDCl_3_) δ 19.0 (CH), 90.6 (CH), 92.4 (C), 114.9 (C), 122.1 (CH), 123.4 (CH), 126.8 (C), 126.9 (CH), 126.9 (C), 127.5 (CH), 132.7 (CH), 139.2 (C), 143.5 (C), 146.2 (C), 146.4 (C) ppm. ^19^F-NMR (376 MHz, CDCl_3_) δ −61.9 ppm. HRMS: calcd for C_15_H_11_F_3_N_5_ [M + H]^+^ 318.0961, found 318.0964.

*5-Methyl-7-(piperidin-1-yl)-[1,2,4]triazolo[1,5-a]pyrimidine* (**7**) [75]. From 7-chloro-5-methyl-[1,2,4]triazolo[1,5-*a*]pyrimidine (50 mg; 0.297 mmol) and piperidine (50 mg; 0.594 mmol), (51 mg, 79%), m.p 153–155 °C. ^1^H-NMR (400 MHz, CDCl_3_) δ 1.71–1.79 (m, 6H), 2.52 (s, 3H), 3.62 (s, 1H), 3.71–3.75 (m, 3H), 6.09 (s, 1H), 8.24 (s, 1H) ppm. ^13^C-NMR (100.6 MHz, CDCl_3_) δ24.2 (CH), 25.1 (2xCH), 25.4 (CH), 49.4 (2xCH), 94.4 (CH), 150.4 (C), 154.0 (CH), 157.3 (C), 164.5 (C) ppm.

*4-(5-Methyl-[1,2,4]triazolo[1,5-a]pyrimidin-7-yl)morpholine* (**8**) [76]. From 7-chloro-5-methyl-[1,2,4]triazolo[1,5-*a*]pyrimidine (50 mg; 0.297 mmol) and morpholine (52 mg; 0.594 mmol), (57 mg, 88%), m.p 164–166 °C. ^1^H-NMR (400 MHz, methanol-*d*_4_) δ 2.55 (s, 3H), 3.90 (s, 8H),6.49 (s, 1H), 8.32 (s, 1H) ppm. ^13^C-NMR (100.6 MHz, methanol-*d*_4_) δ 23.3 (CH), 65.9 (4xCH), 94.4 (CH), 150.4 (C), 152.9 (CH), 156.7 (C), 165.5 (C) ppm.

*(2R,6S)-2,6-Dimethyl-4-(5-methyl-[1,2,4]triazolo[1,5-a]pyrimidin-7-yl)morpholine* (**9**). From 7-chloro-5-methyl-[1,2,4]triazolo[1,5-*a*]pyrimidine (50 mg; 0.297 mmol) and *cis*-2,6-dimethyl-morpholine (68 mg; 0.594 mmol), (65 mg, 89%), m.p 166–168 °C. ^1^H-NMR (250 MHz, CDCl_3_) δ 1.26 (d, *J* = 6.3 Hz, 6H), 2.56 (s, 3H), 2.67–2.76 (m, 2H), 3.84–3.95 (m, 2H), 4.35 (d, *J* = 11.6 Hz, 2H), 6.11 (s, 1H), 8.27 (s, 1H) ppm. ^13^C-NMR (63 MHz, CDCl_3_) δ 18.8 (2xCH), 25.2 (CH), 53.1 (2xCH), 71.2 (2xCH), 94.4 (CH), 149.9 (C), 154.2 (CH), 157.2 (C), 164.9 (C) ppm. CAS: 950030-11-2; Distributor Name: Aurora Fine Chemicals Ltd., A-8010 Graz, Austria. 

*N,N-Di butyl-5-methyl-[1,2,4]triazolo[1,5-a]pyrimidin-7-amine* (**10**). (69 mg, 88%), m.p 130–132 °C. ^1^H-NMR (400 MHz, CDCl_3_) δ 0.94 (t, *J* = 7.4 Hz, 6H), 1.33–1.39 (m, 4H), 1.66 (s, 2H), 2.50 (s, 3H), 3.64 (s, 2H), 3.74–3.78 (m, 4H), 5.90 (s, 1H), 8.18 (s, 1H) ppm. ^13^C-NMR (100.6 MHz, CDCl_3_) δ 13.8 (2xCH), 20.0 (2xCH), 25.1 (CH), 30.1 (CH), 51.6 (2xCH), 70.5 (CH), 92.3 (CH), 149.1 (C), 153.7 (CH), 157.9 (C), 163.7 (C) ppm. HRMS: calcd for C_14_H_24_N_5_ [M + H]^+^ 262.2026, found 262.2027.

*8-(Piperidin-1-yl)-[1,2,4]triazolo[4,3-a]pyrazine* (**11**). From 8-chloro-[1,2,4]triazolo[4,3-*a*]pyrazine (50 mg; 0.323 mmol) and piperidine (55 mg; 0.646 mmol), (61 mg, 92%), m.p 180–182 °C. ^1^H-NMR (400 MHz, CDCl_3_) δ 1.68 (s, 6H), 4.24 (s, 4H), 7.25 (d, *J* = 4.5 Hz, 1H), 7.36 (d, *J* = 4.5 Hz, 1H), 8.68 (s, 1H) ppm. ^13^C-NMR (100.6 MHz, CDCl_3_) δ 24.7 (CH), 26.2 (2xCH), 47.4 (2xCH), 106.0 (CH), 129.8 (CH), 136.6 (CH), 140.5 (C), 147.9 (C) ppm. CAS:1878022-44-6; Distributor Name: Sigma-Aldrich, F 38297 Saint-Quentin Fallavier, France.

*(2R,6S)-4-([1,2,4]Triazolo[4,3-a]pyrazin-8-yl)-2,6-dimethylmorpholine* (**12**). From 8-chloro-[1,2,4]-triazolo[4,3-*a*]pyrazine (50 mg; 0.323 mmol) and *cis*-2,6-dimethyl-morpholine (74 mg; 0.646 mmol), (74 mg, 99%), m.p 172–174 °C. ^1^H-NMR (400 MHz, CDCl_3_) δ 1.22 (d, *J* = 6.3 Hz, 6H), 2.73–2.81 (m, 2H), 3.57–3.59 (m, 1H), 3.66–3.73 (m, 2H), 5.33 (s, 1H), 7.27 (d, *J* = 4.5 Hz, 1H), 7.41 (d, *J* = 4.5 Hz, 1H), 8.71 (s, 1H) ppm. ^13^C-NMR (100.6 MHz, CDCl_3_) δ 18.8 (2xCH), 71.9 (2xCH), 106.8 (CH), 129.5 (CH), 136.7 (CH), 140.3 (C), 147.7 (2xCH), 160.6 (C) ppm. CAS: 2127319-44-0; Distributor Name: Aurora Fine Chemicals Ltd., A-8010 Graz, Austria.

*N,N-Dibutyl-[1,2,4]triazolo[4,3-a]pyrazin-8-amine* (**13**). From 8-chloro-[1,2,4]triazolo[4,3-*a*]pyrazine (50 mg; 0.323 mmol) and di-*n*-butylamine (83 mg; 0.646 mmol),(58 mg, 73%), m.p 106–108 °C. ^1^H-NMR (400 MHz, CDCl_3_) δ 0.92 (t, *J* = 7.4 Hz, 6H), 1.34–1.41 (m, 4H), 1.67 (t, *J* = 7.7 Hz, 4H), 4.00 (s, 4H), 7.27 (d, *J* = 4.5 Hz, 1H), 7.32 (d, *J* = 4.5 Hz, 1H), 8.67 (s, 1H) ppm. ^13^C-NMR (100.6 MHz, CDCl_3_) δ14.0 (3xCH), 20.1 (2xCH), 30.4 (CH), 49.5 (2xCH), 105.3 (CH), 130.1 (CH), 136.5 (CH), 140.4 (C), 148.0 (C) ppm. HRMS: calcd for C_13_H_22_N_5_ [M + H]^+^ 248.1870, found 248.1871.

*N-(2-(Trifluoromethyl)phenyl)-[1,2,4]triazolo[4,3-a]pyrazin-8-amine* (**14**). From 8-chloro-[1,2,4]triazolo-[4,3-*a*]pyrazine (50 mg; 0.323 mmol) and 2-trifluoromethylaniline (104 mg; 0.646 mmol), (68 mg, 75%), m.p 169–171 °C. ^1^H-NMR (400 MHz, CDCl_3_) δ 7.29 (d, *J* = 7.6 Hz, 1H), 7.44 (d, *J* = 4.7 Hz, 1H), 7.60 (d, *J* = 4.7 Hz, 1H), 7.63 (t, *J* = 7.9 Hz, 1H), 7.71 (d, *J* = 7.8 Hz, 1H), 8.39 (s, 1H), 8.53 (d, *J* = 8.3 Hz, 1H), 8.82 (s, 1H) ppm. ^13^C-NMR (100.6 MHz, CDCl_3_) δ109.2 (CH), 121.0 (C), 124.3 (2xCH), 125.4 (C), 126.6 (CH), 129.2 (CH), 132.7 (CH), 135.6 (C), 137.4 (CH), 139.2 (C), 145.4 (C) ppm. ^19^F-NMR (376 MHz, CDCl_3_) δ −60.7 ppm. HRMS: calcd for C_12_H_9_F_3_N_5_ [M + H]^+^ 280.0805, found 280.0805.

*4-(Piperidin-1-yl)furo[3,2-d]pyrimidine* (**15**). From 4-chlorofuro[3,2-*d*]pyrimidine (50 mg; 0.323 mmol) and piperidine (55 mg; 0.646 mmol), (65 mg, 99%), m.p 140–142 °C. ^1^H-NMR (400 MHz, CDCl_3_) δ 1.64–1.73 (m, 6H), 3.94–3.98 (m, 4H), 6.80 (d, *J* = 2.2 Hz, 1H), 7.68 (d, *J* = 2.2 Hz, 1H), 8.41 (s, 1H) ppm.^13^C-NMR (100.6 MHz, CDCl_3_) δ 24.7 (CH),26.0 (2xCH), 46.3 (2xCH), 107.8 (CH), 134.4 (C), 147.1 (CH), 148.4 (C), 151.0 (C), 153.4 (CH) ppm. HRMS: calcd forC_11_H_14_N_3_O [M + H]^+^ 204.1131, found 204.1135.

*4-(Piperidin-1-yl)thieno[3,2-d]pyrimidine* (**16**). From 4-chlorothieno[3,2-*d*]pyrimidine (50 mg; 0.293 mmol) and piperidine (50 mg; 0.586 mmol), (55 mg, 86%), m.p 154–156 °C. ^1^H-NMR (400 MHz, CDCl_3_) δ 1.10–1.61 (m, 6H), 3.87–3.90 (m, 4H), 7.33 (d, *J* = 5.6 Hz, 1H), 7.62 (d, *J* = 5.6 Hz, 1H), 8.49 (s, 1H) ppm. ^13^C-NMR (100.6 MHz, CDCl_3_) δ 24.7 (CH), 26.1 (2xCH), 47.4 (2xCH), 114.2 (C), 125.1 (CH), 131.1 (CH), 154.2 (CH), 157.8 (C), 161.0 (C) ppm. CAS: 679394-37-7; Distributor Name: SIA Enamine, LV-1035 Riga, Latvia.

*4-Morpholinofuro[3,2-d]pyrimidine* (**17**). From 4-chlorofuro[3,2-*d*]pyrimidine (50 mg; 0.323 mmol) and morpholine (56 mg; 0.646 mmol), (65 mg, 99%), m.p 185–187 °C. ^1^H-NMR (400 MHz, CDCl_3_) δ 3.81–3.85 (m, 4H), 4.00–4.04 (m, 4H), 6.84 (d, *J* = 2.2 Hz, 1H), 7.72 (d, *J* = 2.2 Hz, 1H), 8.46 (s, 1H) ppm. ^13^C-NMR (101 MHz, CDCl_3_) δ 45.5 (2xCH), 66.84 (2xCH), 107.96 (CH), 134.3 (C), 147.6 (CH), 148.43 (C), 151.5 (C), 153.3 (CH) ppm. HRMS: calcd for C_10_H_12_N_3_O_2_ [M + H]^+^ 206.0924, 206.0926 found.

*4-(Thieno[3,2-d]pyrimidin-4-yl)morpholine* (**18**) [77]. From 4-chlorothieno[3,2-*d*]pyrimidine (50 mg; 0.293 mmol) and morpholine (51 mg; 0.586 mmol), (58 mg, 90%), m.p 141–143 °C. ^1^H-NMR (400 MHz, CDCl_3_) δ 3.78–3.82 (m, 4H), 3.91–3.97 (m, 4H), 7.39 (d, *J* = 5.6 Hz, 1H), 7.69 (d, *J* = 5.6 Hz, 1H), 8.56 (s, 1H) ppm. ^13^C-NMR (100.6 MHz, CDCl_3_) δ 46.3 (2xCH), 66.7 (2xCH), 114.4 (C), 125.3 (CH), 131.5 (CH), 154.2 (CH), 158.2 (C), 161.6 (C) ppm.

*(2R,6S)-2,6-Dimethyl-4-(thieno[3,2-d]pyrimidin-4-yl)morpholine* (**19**). From 4-chlorothieno[3,2-*d*]pyrimidine (50 mg; 0.293 mmol) and *cis*-2,6-dimethyl-morpholine (67 mg; 0.586 mmol), (60 mg, 83%), m.p 142–144 °C. ^1^H ^1^H-NMR (400 MHz, CDCl_3_) δ 1.25 (d, *J* = 6.3 Hz, 6H), 2.79–2.85 (m, 2H), 3.61–3.73 (m, 2H), 4.60 (d, *J* = 13.0 Hz, 2H), 7.39 (d, *J* = 5.6 Hz, 1H), 7.69 (d, *J* = 5.6 Hz, 1H), 8.54 (s, 1H) ppm. ^13^C-NMR (100.6 MHz, CDCl_3_) δ18.8 (2xCH), 51.3 (2xCH), 71.8 (2xCH), 114.3 (C), 125.2 (CH), 131.5 (CH), 154.1 (CH), 157.8 (C), 161.3 (C) ppm. CAS: 676119-22-5; Distributor Name: Aurora Fine Chemicals Ltd., A-8010 Graz, Austria.

*N,N-Dibutylthieno[3,2-d]pyrimidin-4-amine* (**20**). From 4-chlorothieno[3,2-*d*]pyrimidine (50 mg; 0.293 mmol) and di-*n*-butylamine (76 mg; 0.586 mmol), (74 mg, 96%), m.p 121–123 °C. ^1^H-NMR (400 MHz, CDCl_3_) δ 0.92 (t, *J* = 7.4 Hz, 6H), 1.32–1.40 (m, 4H), 1.64 (dd, *J* = 6.6, 16.9 Hz, 4H), 3.64–3.68 (m, 4H), 7.31 (d, *J* = 5.6 Hz, 1H), 7.62 (d, *J* = 5.6 Hz, 1H), 8.45 (s, 1H) ppm. ^13^C-NMR (100.6 MHz, CDCl_3_) δ 13.9 (2xCH), 20.1 (2xCH), 30.8 (2xCH), 49.3 (2xCH), 113.3 (C), 124.9 (CH), 130.9 (CH), 154.3 (CH), 157.5 (C), 160.6 (C) ppm. HRMS: calcd for C_14_H_22_N_3_S [M + H]^+^ 264.1529, found 264.1532.

*N-((3s,5s,7s)-Adamantan-1-yl)thieno[3,2-d]pyrimidin-4-amine* (**21**). From 4-chlorothieno[3,2-*d*]-pyrimidine (50 mg; 0.293 mmol) and adamantylamine (88 mg; 0.586 mmol), (64 mg, 77%), m.p 238–240 °C. ^1^H-NMR (400 MHz, CDCl_3_) δ 1.70–1.77 (m, 6H), 2.14 (s, 3H), 2.24 (d, *J* = 2.6 Hz, 6H), 4.52 (s, 1H), 7.36 (d, *J* = 5.4 Hz, 1H), 7.61 (d, *J* = 5.4 Hz, 1H), 8.57 (s, 1H) ppm. ^13^C-NMR (100.6 MHz, CDCl_3_) δ29.6 (3xCH), 36.4 (3xCH), 41.9 (3xCH), 53.6 (C), 115.6 (C), 125.6 (CH), 129.7 (CH), 154.6 (CH), 156.9 (C), 159.5 (C) ppm. HMRS: calcd for C_16_H_20_N_3_S [M + H]^+^ 286.1372, found 286.1375.

*N-(2-(Trifluoromethyl)phenyl)thieno[3,2-d]pyrimidin-4-amine* (**22**). From 4-chlorothieno[3,2-*d*]-pyrimidine (50 mg; 0.293 mmol) and 2-trifluoromethylaniline(94 mg; 0.586 mmol), (61 mg, 71%), m.p 122–124 °C. ^1^H-NMR (400 MHz, CDCl_3_) δ7.37 (t, *J* = 7.7 Hz, 1H), 7.45 (d, *J* = 5.4 Hz, 1H), 7.62 (t, *J* = 7.8 Hz, 1H), 7.71–7.75 (m, 2H), 7.99 (d, *J* = 8.1 Hz, 1H), 8.70 (s, 1H), ppm. ^13^C-NMR (100.6 MHz, CDCl_3_) δ122.5 (C), 124.2 (C), 124.5 (C), 125.1 (CH), 126.0 (CH), 126.7 (CH), 127.8 (CH), 132.6 (CH), 132.9 (CH), 135.3 (C), 154.5 (CH), 155.9 (C), 161.5 (C) ppm. ^19^F-NMR (376 MHz, CDCl_3_) δ −60.8 ppm. HRMS: calcd for C_13_H_9_F_3_N_3_S [M + H]^+^ 296.0464, found 296.0467.

*Methyl 4-(thieno[3,2-d]pyrimidin-4-ylamino)benzoate* (**23**). From 4-chlorothieno[3,2-*d*]pyrimidine (50 mg; 0.293 mmol) and methyl 4-aminobenzoate (88 mg; 0.586 mmol), (66 mg, 79%), m.p 227–229 °C. ^1^H-NMR (400 MHz, CDCl_3_) δ 3.93 (s, 3H), 6.97 (d, *J* = 19.8 Hz, 1H), 7.51 (d, *J* = 5.4 Hz, 1H), 7.84–7.79 (m, 3H), 8.09 (d, *J* = 8.7 Hz, 2H), 8.82 (s, 1H) ppm. ^13^C-NMR (100.6 MHz, CDCl_3_) δ 29.7 (C), 52.1 (CH), 116.2 (C), 120.5 (2xCH), 125.6 (CH), 130.9 (2xCH), 132.1 (CH), 142.4 (C), 154.6 (CH), 154.9 (C), 161.2 (C), 166.6 (C) ppm. HRMS: calcd forC_14_H_12_N_3_O_2_S [M + H] ^+^ 286.0644, found 286.0646.

*N-(4-Methoxyphenyl)thieno[3,2-d]pyrimidin-4-amine* (**24**) [78]. From 4-chlorothieno[3,2-*d*]pyrimidine (50 mg; 0.293 mmol) and methyl 4-methoxyaniline (72 mg; 0.586 mmol),(66 mg, 88%), m.p 154–156 °C. ^1^H-NMR (400 MHz, CDCl_3_) δ 3.85 (s, 3H), 6.93–6.95 (m, 2H), 7.36 (d, *J* = 5.4 Hz, 1H), 7.39–7.41 (m, 2H), 7.64 (d, *J* = 5.4 Hz, 1H), 7.93 (s, 1H), 8.60 (s, 1H) ppm. ^13^C-NMR (100.6 MHz, CDCl_3_) δ 55.5 (CH), 114.3 (2xCH), 114.4 (C), 124.6 (2xCH), 128.2 (CH), 129.7 (C), 133.2 (CH), 154.6 (CH), 157.3 (C), 158.6 (C), 161.2 (C) ppm.

## 4. Conclusions

We have developed an efficient, environmentally sound method for the nucleophilic aromatic substitution in PEG400 of chlorine atoms by primary and secondary amines on various nitrogen-containing fused heterocycles. The salient feature of our method is the facile introduction of amino derivatives on commercially available starting materials in an environmentally friendly alternative solvent. Several precursors of potential biologically active compounds have been synthetized in good to excellent yields and the conditions used are applicable to a large panel of heterocycles and amines, with similar yields and reaction time.

## Figures and Tables

**Table 1:** Results of the S_N_Ar on 4-chloro-2-methylimidazo[1,5-*a*]pyrimidine-8-carbonitrile with various amines.

Entry	Amine Reagent	Product	Yield
1	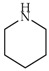	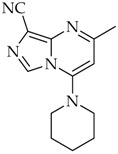	**1**; 87%
2	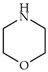	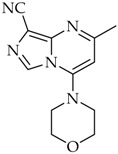	**2**; 92%
3	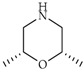	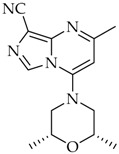	**3**; 95%
4	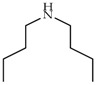	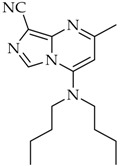	**4**; 81%
5	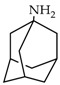	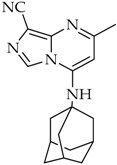	**5**; 85%
6	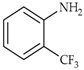	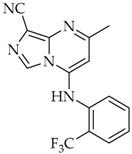	**6**; 70%

**Table 2:** Results of S_N_Ar on 7-chloro-5-methyl-[1,2,4]triazolo[1,5-*a*]pyrimidine with various amines.

Entry	Amine Reagent	Product	Yield
1	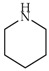	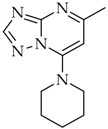	**7**; 79%
2	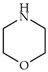	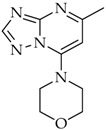	**8**; 88%
3	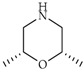	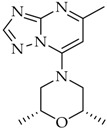	**9**; 89%
4	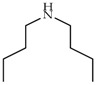	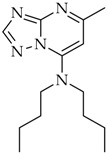	**10**; 88%

**Table 3:** Results of the S_N_Ar on 8-chloro-[1,2,4]triazolo[4,3-*a*]pyrazine with various amines.

Entry	Amine Reagent	Product	Yield
1	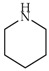	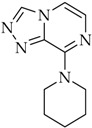	**11**; 92%
2	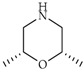	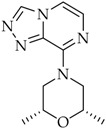	**12**; 99%
3	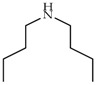	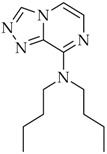	**13**; 73%
4	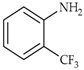	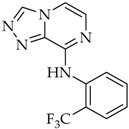	**14**; 75%

**Table 4:** Results of the aromatic nucleophilic substitution (SNAr) on 4-chlorofuro and thieno[3,2-*d*]pyrimidine.

Entry	Amine Reagent	Product	Yield
1	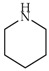	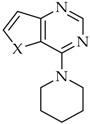	**15**; X = O, 99% **16**; X = S, 86%
2	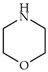	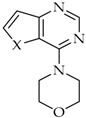	**17**; X = O, 99% **18**; X = S, 90%
3	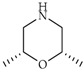	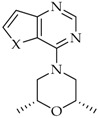	**19**; X = S, 83%
4	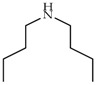	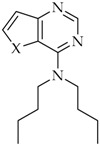	**20**; X= S, 96%
5	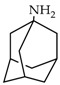	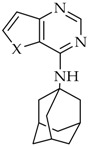	**21**; X = S, 77%
6	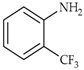	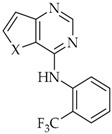	**22**; X= S, 71%
7	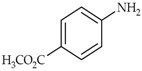	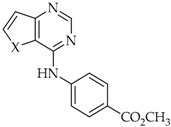	**23**; X = S, 79%
8	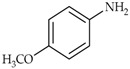	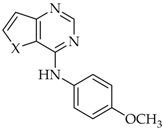	**24**; X = S, 88%

## Supplementary Materials

Supplementary File 1
