# Supplementary material for: A Greener and Efficient Method for Nucleophilic Aromatic Substitution of Nitrogen-Containing Fused Heterocycles
Source: Molecules. 2018 Mar 18;23(3):684. doi: 10.3390/molecules23030684 (PMC6017854; doi:10.3390/molecules23030684)
Supplement: Supplementary File 1 [file molecules-23-00684-s001.pdf]

molecules

MDPI

Article

A green and efficient method for Nucleophilic Aromatic substitution of Nitrogen-containing fused heterocycles

Joana F. Campos [1], Mohammed Loubidi [1], Marie-Christine Scherrmann[2] and

Sabine Berteina-Raboin[1,*]11

[1] Institut de Chimie Organique et Analytique (ICOA), Université d'Orléans, UMR-CNRS 7311, BP 6759, rue de Chartres, France

[2] Institut de Chimie Moléculaire et des Matériaux d'Orsay, UMR CNRS 8182, Université Paris-Sud, Bâtiment 420, 91400 Orsay, France

* Correspondence: sabine.berteina-raboin@univ- orleans.fr; Tel.: +33-238-494-856

Academic Editor: Thierry Besson

Received: 27 February 2018; Accepted: 16 March 2018; Published: 18 March 2018

Compound 1

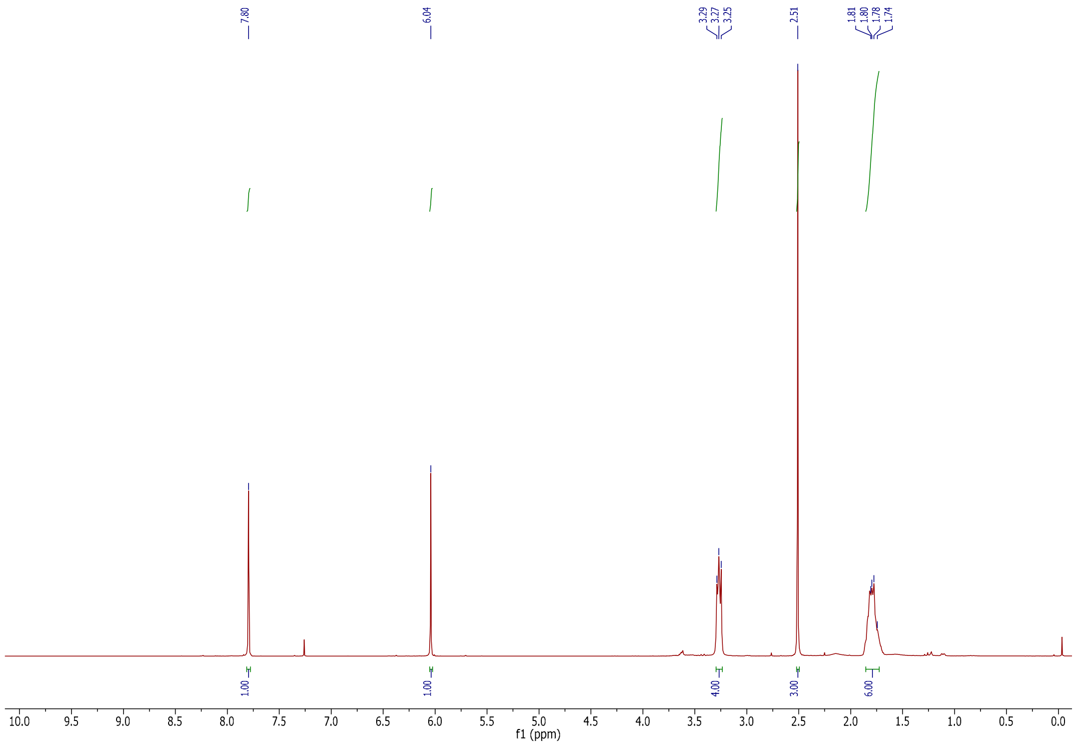

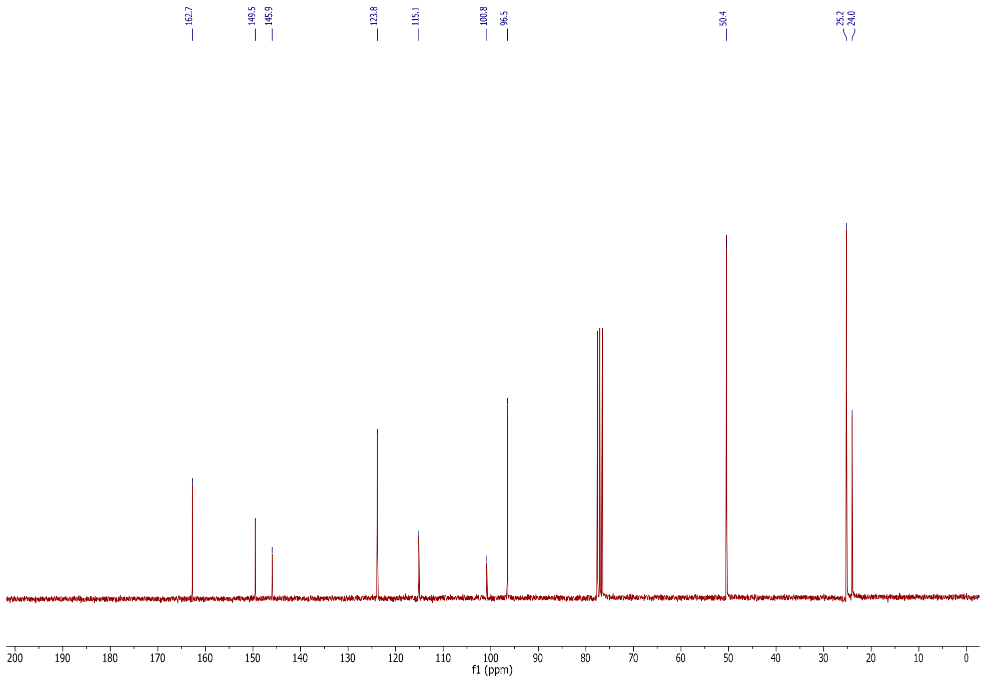

162.7
149.5
145.9
123.8
115.1
100.8
96.5
50.4
25.2
24.0
200 190 180 170 160 150 140 130 120 110 100 90 80 70 60 50 40 30 20 10 0
f1 (ppm)

Compound 2

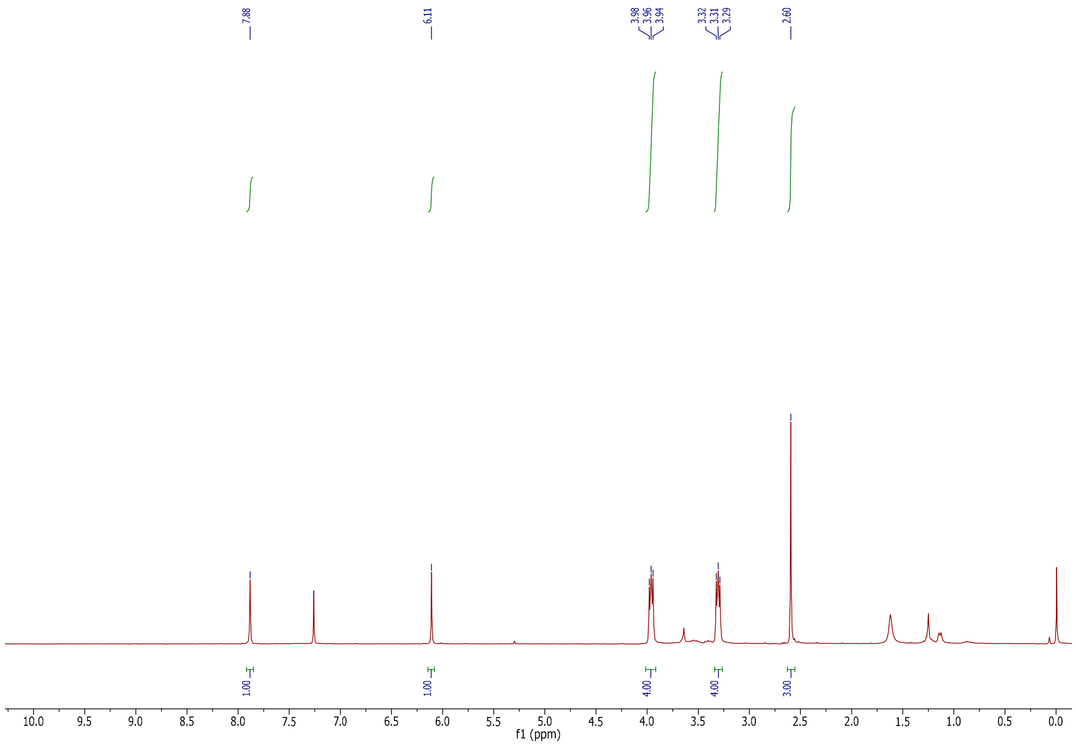

7.88
6.11
3.98
3.96
3.94
3.32
3.31
3.29
2.60
1.00
1.00
4.00
4.00
3.00
10.0 9.5 9.0 8.5 8.0 7.5 7.0 6.5 6.0 5.5 5.0 4.5 4.0 3.5 3.0 2.5 2.0 1.5 1.0 0.5 0.0
f1 (ppm)

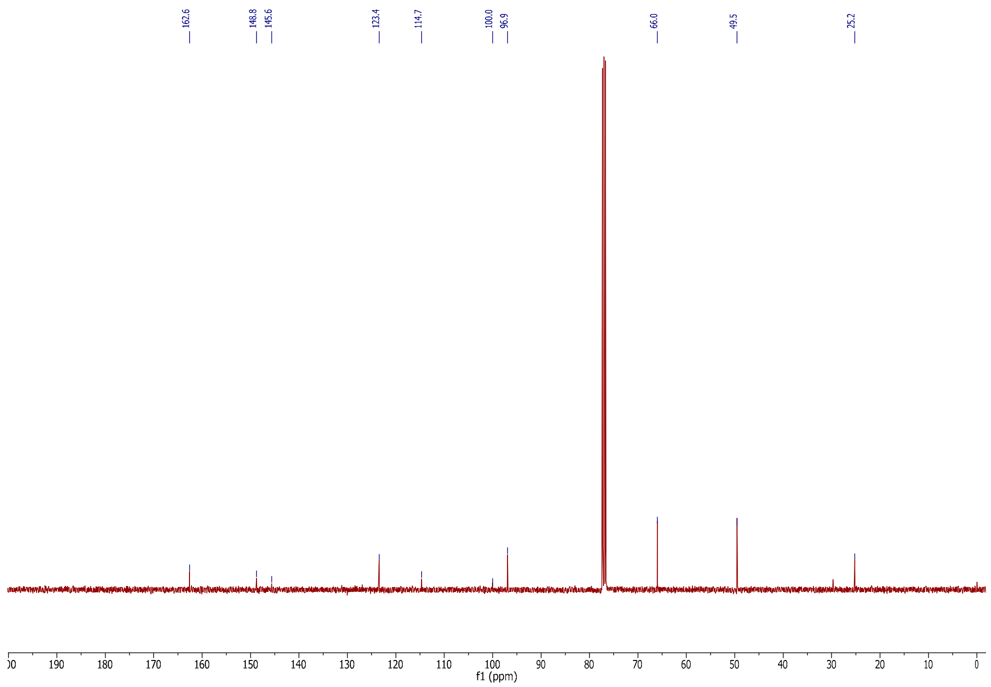
162.6
148.8
145.6
123.4
114.7
100.0
96.9
66.0
49.5
25.2
f1 (ppm)

Compound 3

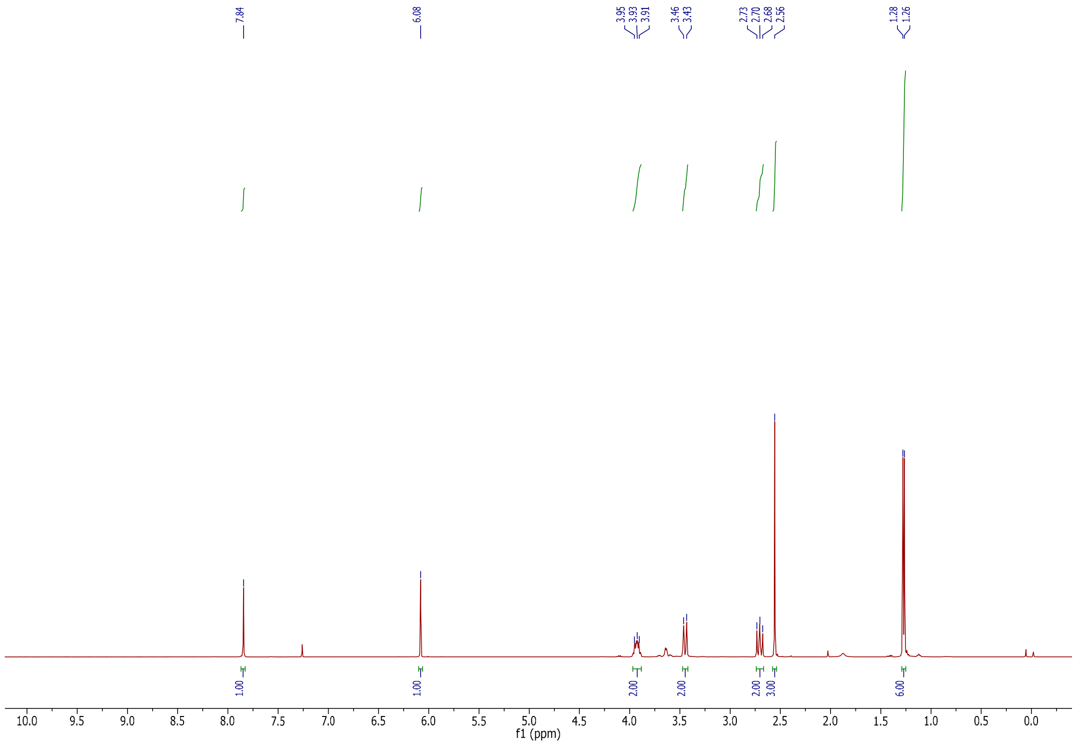
7.84
6.08
3.95
3.93
3.91
3.46
3.43
2.73
2.70
2.68
2.56
1.28
1.26
1.00
1.00
2.00
2.00
2.00
3.00
6.00
f1 (ppm)

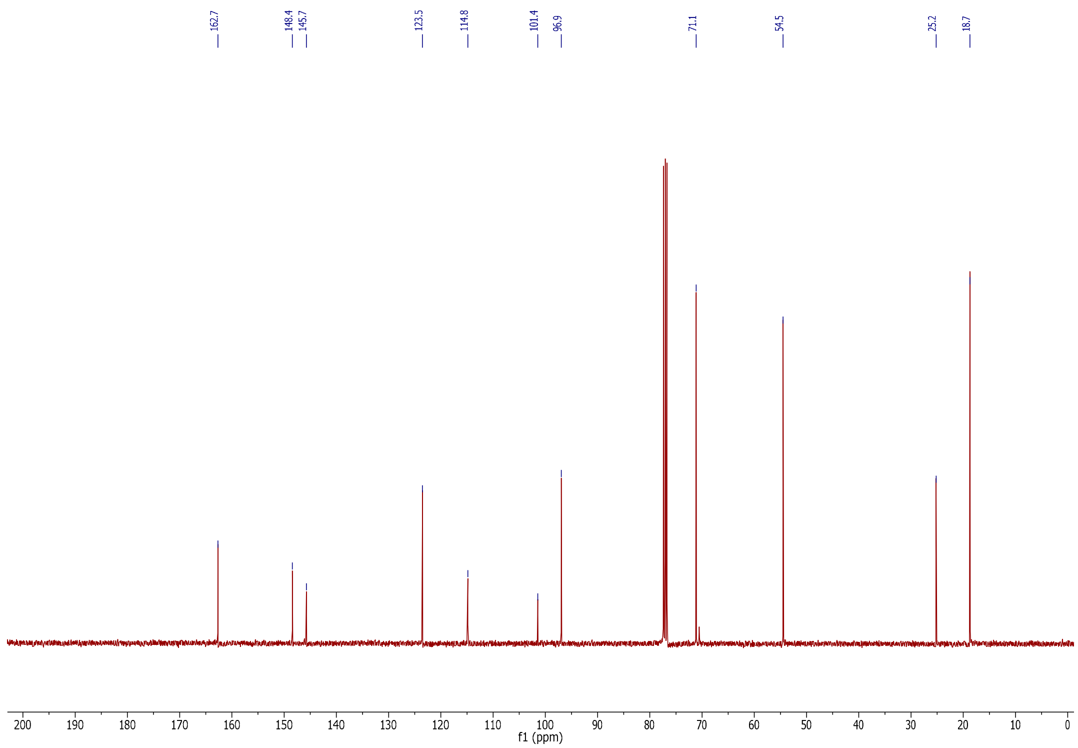
162.7
148.4
145.7
123.5
114.8
101.4
96.9
71.1
54.5
25.2
18.7
f1 (ppm)

Compound 4

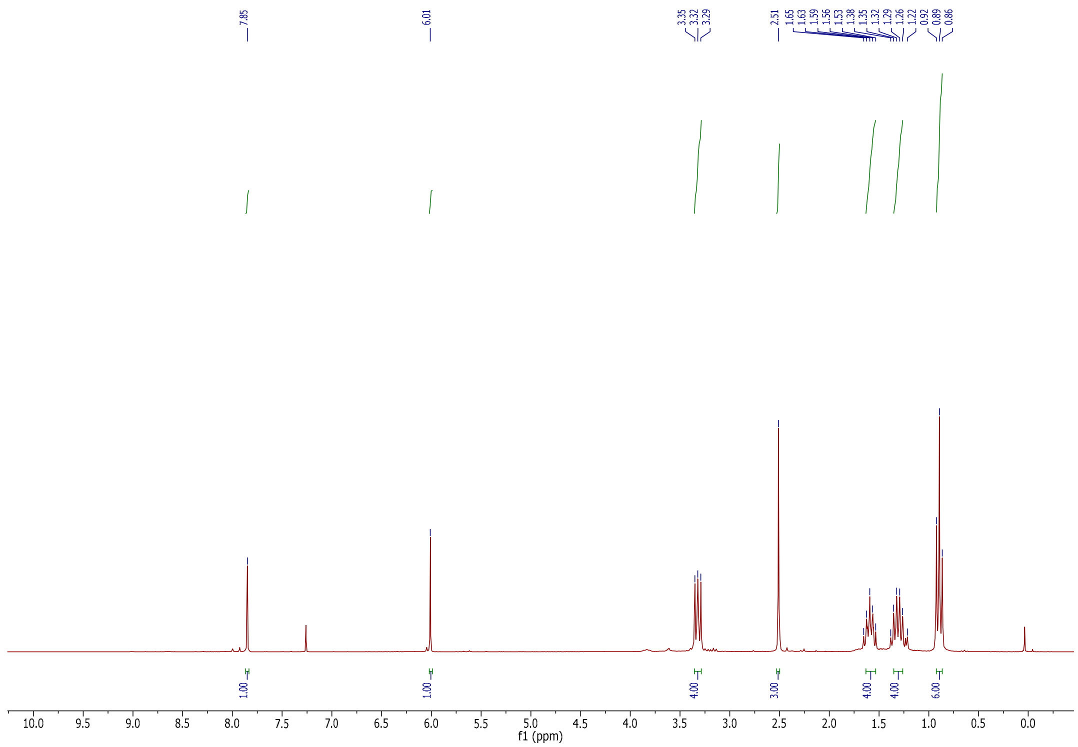
7.85
6.01
3.35
3.32
3.29
2.51
1.65
1.63
1.59
1.56
1.53
1.38
1.35
1.32
1.29
1.26
1.22
0.92
0.89
0.86
1.00
1.00
4.00
3.00
4.00
4.00
6.00
f1 (ppm)

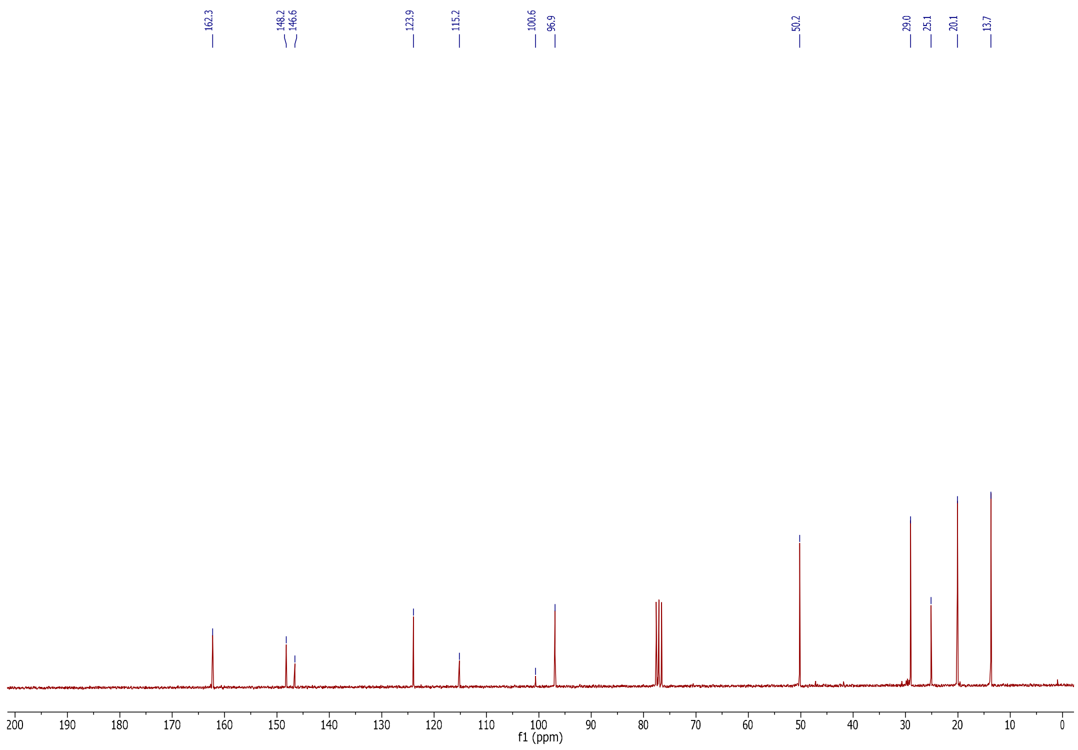
162.3
148.2
146.6
123.9
115.2
100.6
96.9
50.2
29.0
25.1
20.1
13.7
f1 (ppm)

Compound 5

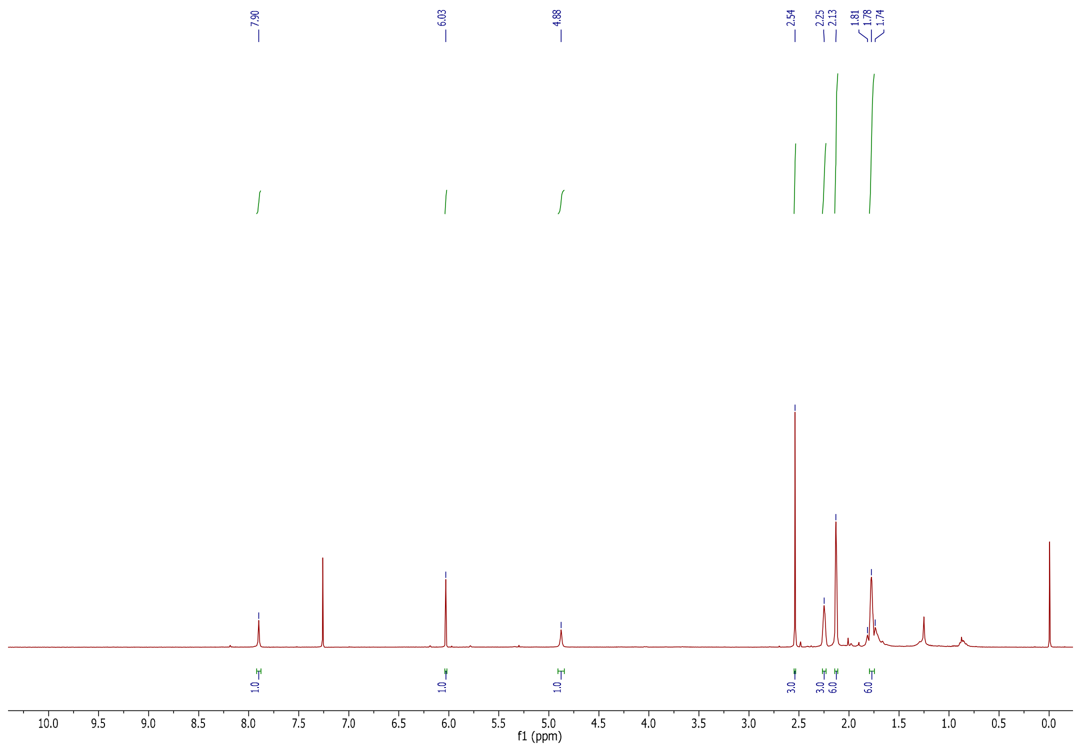
7.90
6.03
4.88
2.54
2.25
2.13
1.81
1.78
1.74
1.0
1.0
1.0
3.0
3.0
6.0
6.0
f1 (ppm)

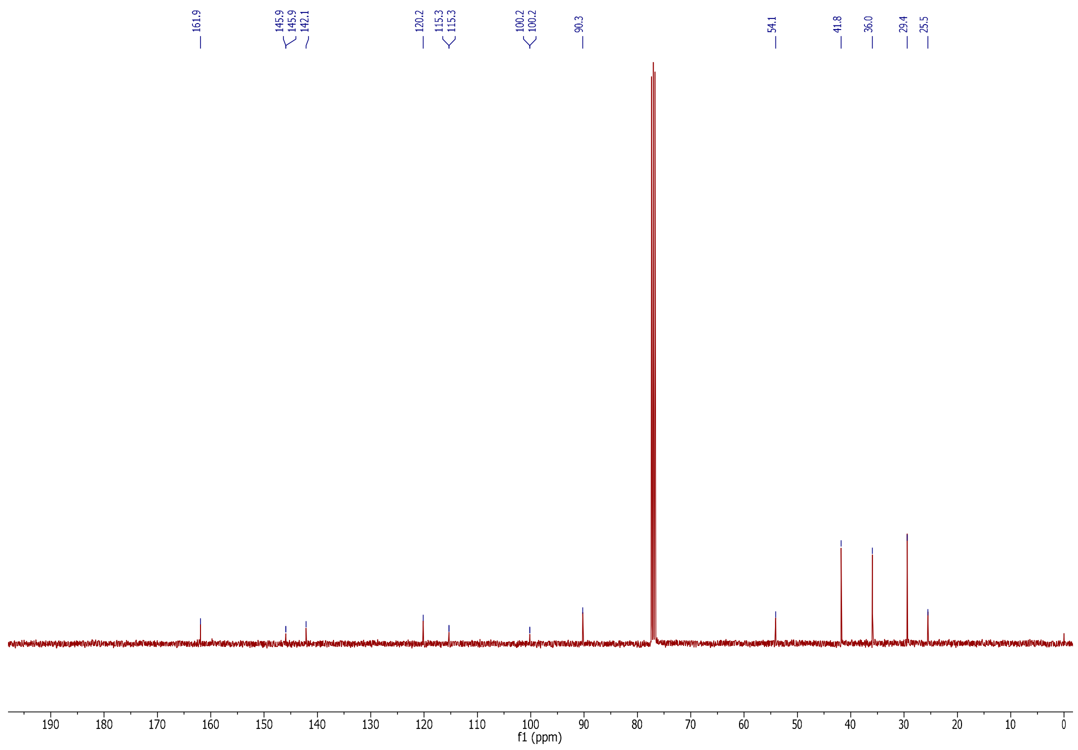
161.9
145.9
145.9
142.1
120.2
115.3
115.3
100.2
100.2
90.3
54.1
41.8
36.0
29.4
25.5
f1 (ppm)

Compound 6

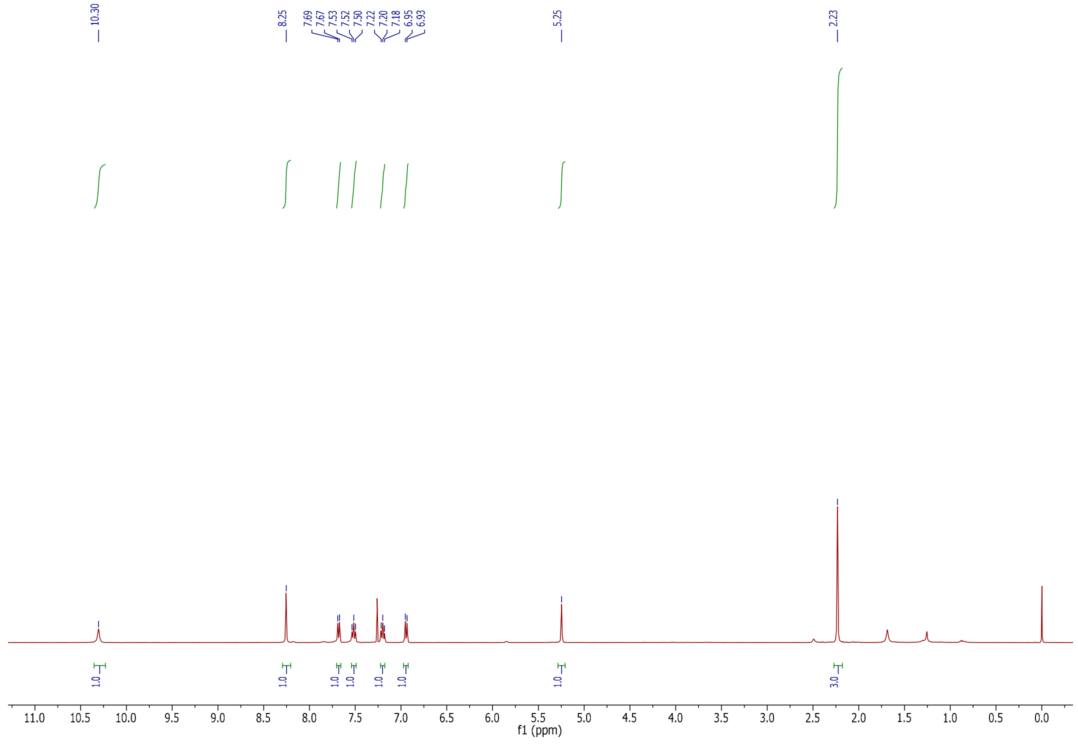
10.30
8.25
7.69
7.67
7.53
7.52
7.50
7.22
7.20
7.18
6.95
6.93
5.25
2.23
1.0
1.0
1.0
1.0
1.0
1.0
1.0
3.0
f1 (ppm)

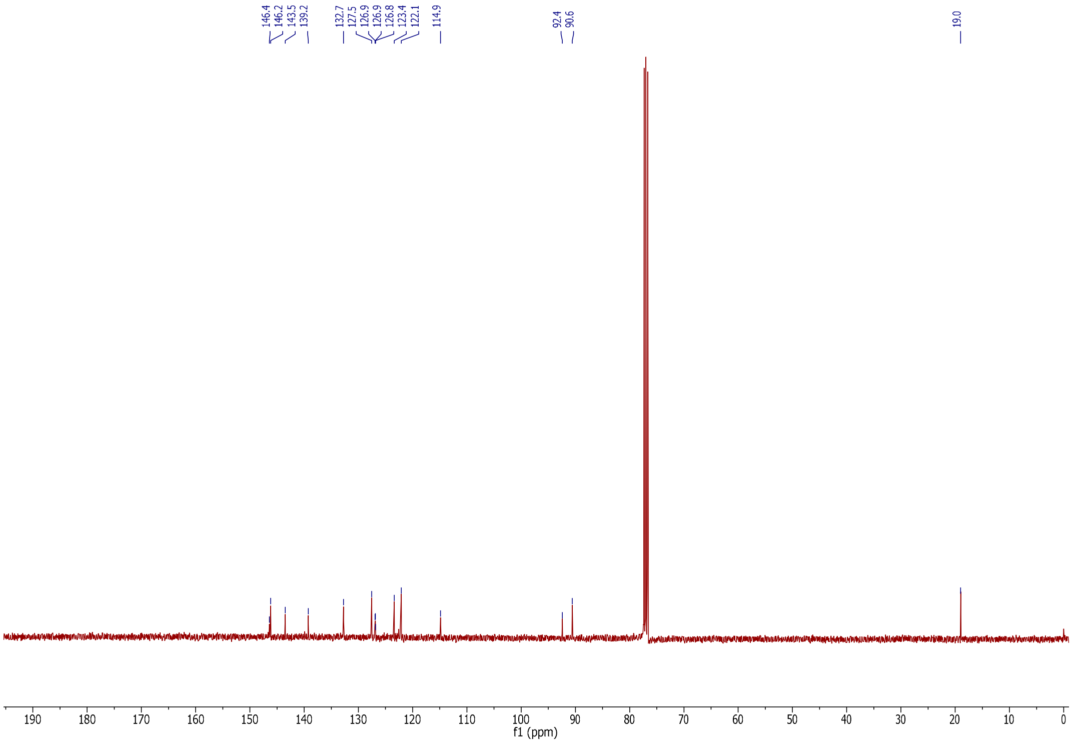
146.4
146.2
143.5
139.2
132.7
127.5
126.9
126.9
126.8
123.4
122.1
114.9
92.4
90.6
19.0
190 180 170 160 150 140 130 120 110 100 90 80 70 60 50 40 30 20 10 0
f1 (ppm)

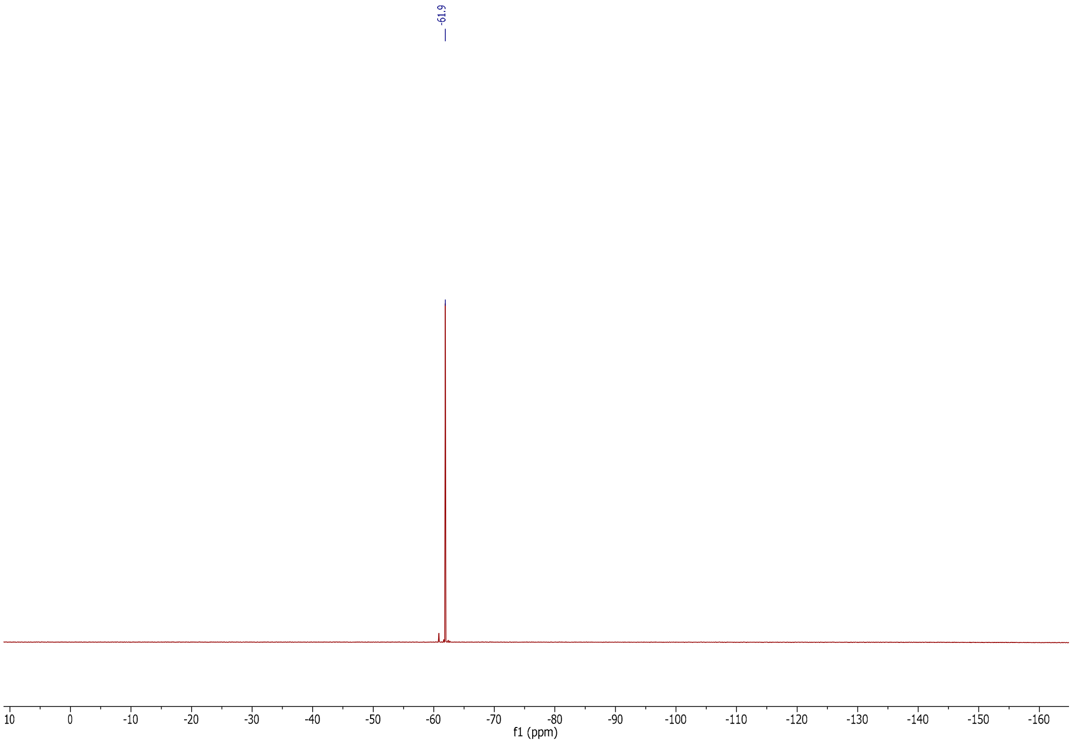
-61.9
10 0 -10 -20 -30 -40 -50 -60 -70 -80 -90 -100 -110 -120 -130 -140 -150 -160
f1 (ppm)

Compound 7

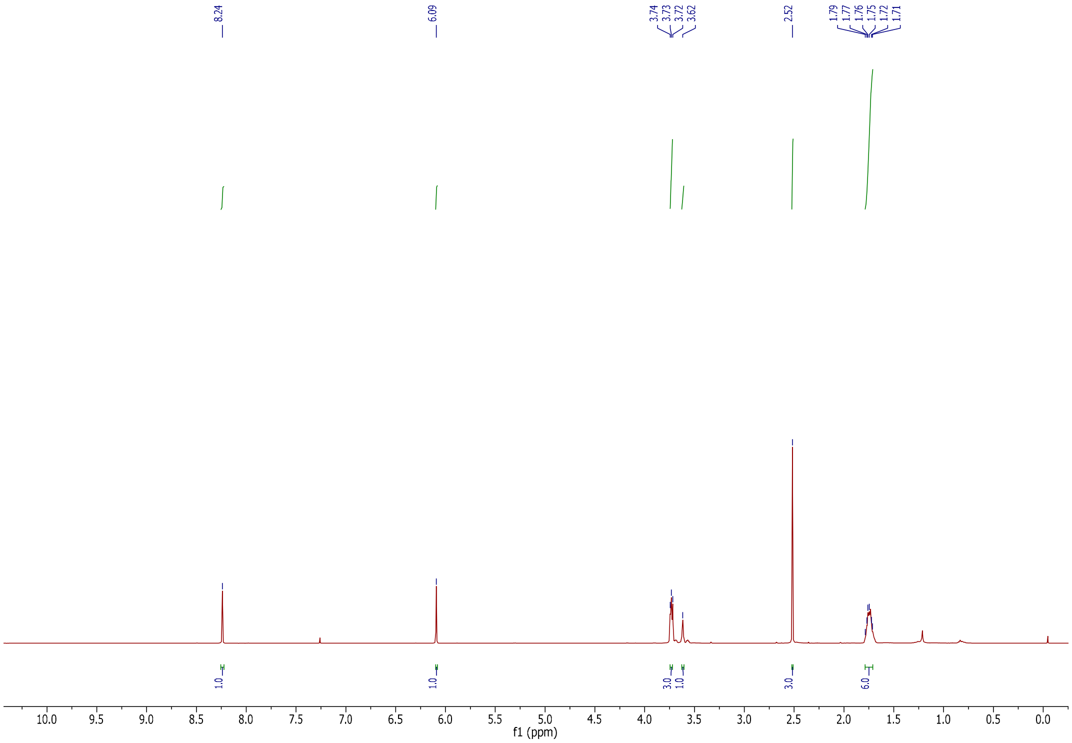
8.24
6.09
3.74
3.73
3.72
3.62
2.52
1.79
1.77
1.76
1.75
1.72
1.71
1.0
1.0
3.0
1.0
3.0
6.0
10.0 9.5 9.0 8.5 8.0 7.5 7.0 6.5 6.0 5.5 5.0 4.5 4.0 3.5 3.0 2.5 2.0 1.5 1.0 0.5 0.0
f1 (ppm)

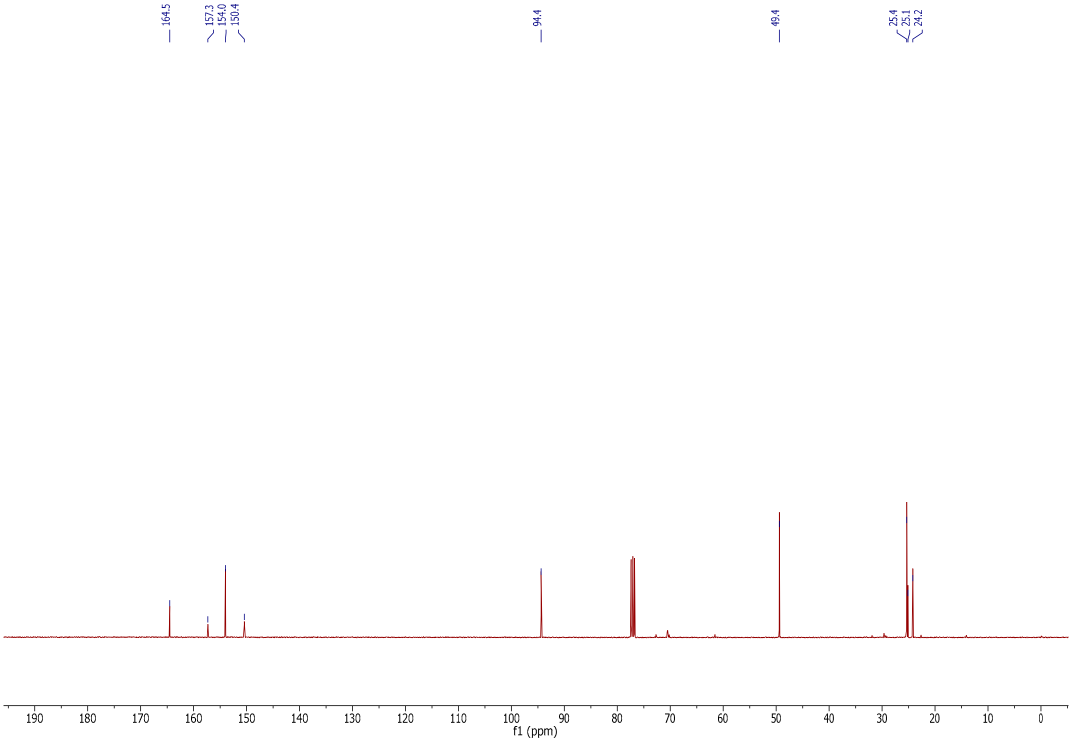
164.5
157.3
154.0
150.4
94.4
49.4
25.4
25.1
24.2
190 180 170 160 150 140 130 120 110 100 90 80 70 60 50 40 30 20 10 0
f1 (ppm)

Compound 8

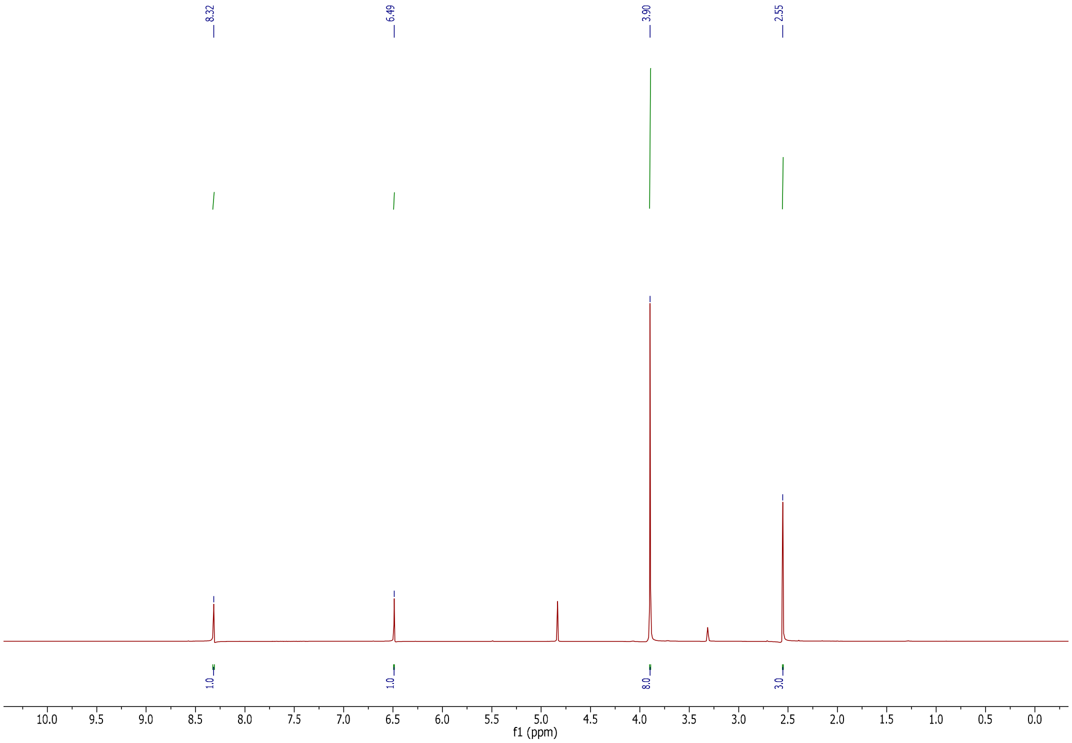
8.32
6.49
3.90
2.55
1.0
1.0
8.0
3.0
10.0 9.5 9.0 8.5 8.0 7.5 7.0 6.5 6.0 5.5 5.0 4.5 4.0 3.5 3.0 2.5 2.0 1.5 1.0 0.5 0.0
f1 (ppm)

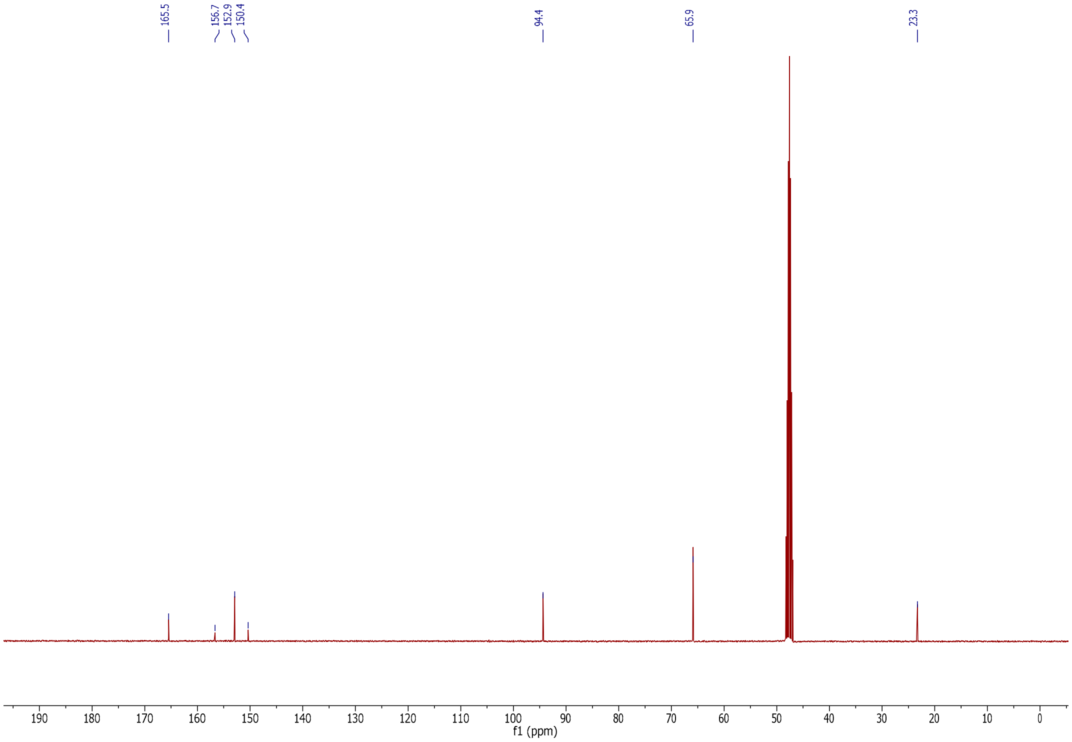
165.5
156.7
152.9
150.4
94.4
65.9
23.3
190 180 170 160 150 140 130 120 110 100 90 80 70 60 50 40 30 20 10 0
f1 (ppm)

Compound 9

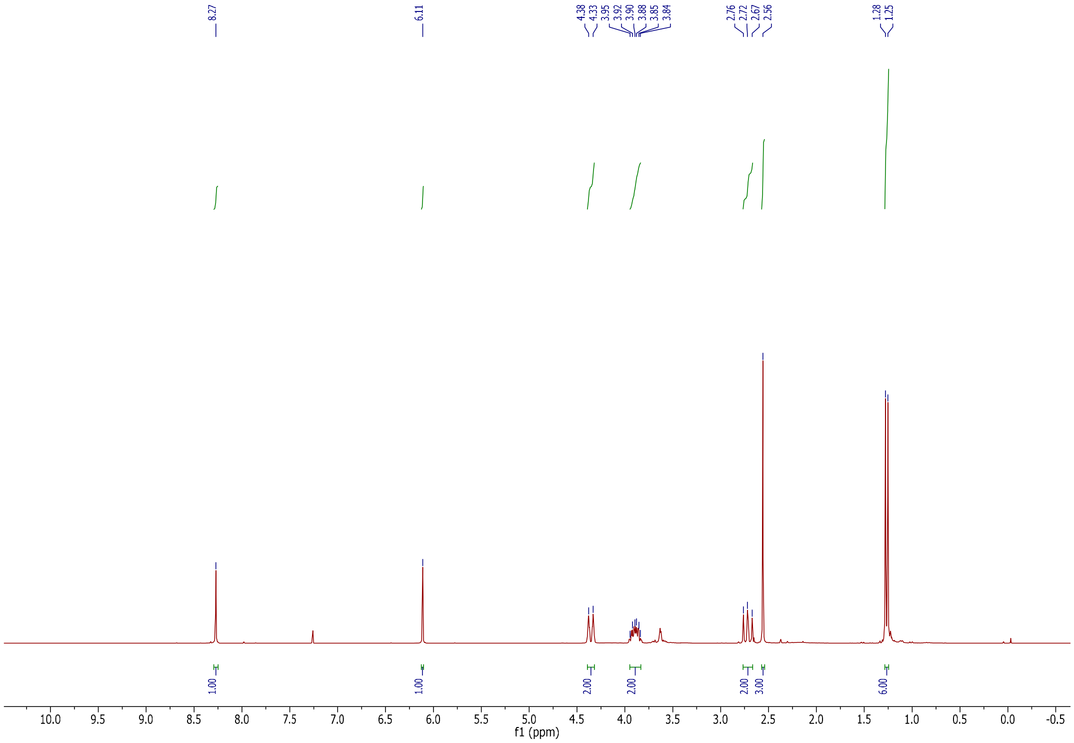
8.27
6.11
4.38
4.33
3.95
3.92
3.90
3.88
3.85
3.84
2.76
2.72
2.67
2.56
1.28
1.25
1.00
1.00
2.00
2.00
2.00
3.00
6.00
f1 (ppm)

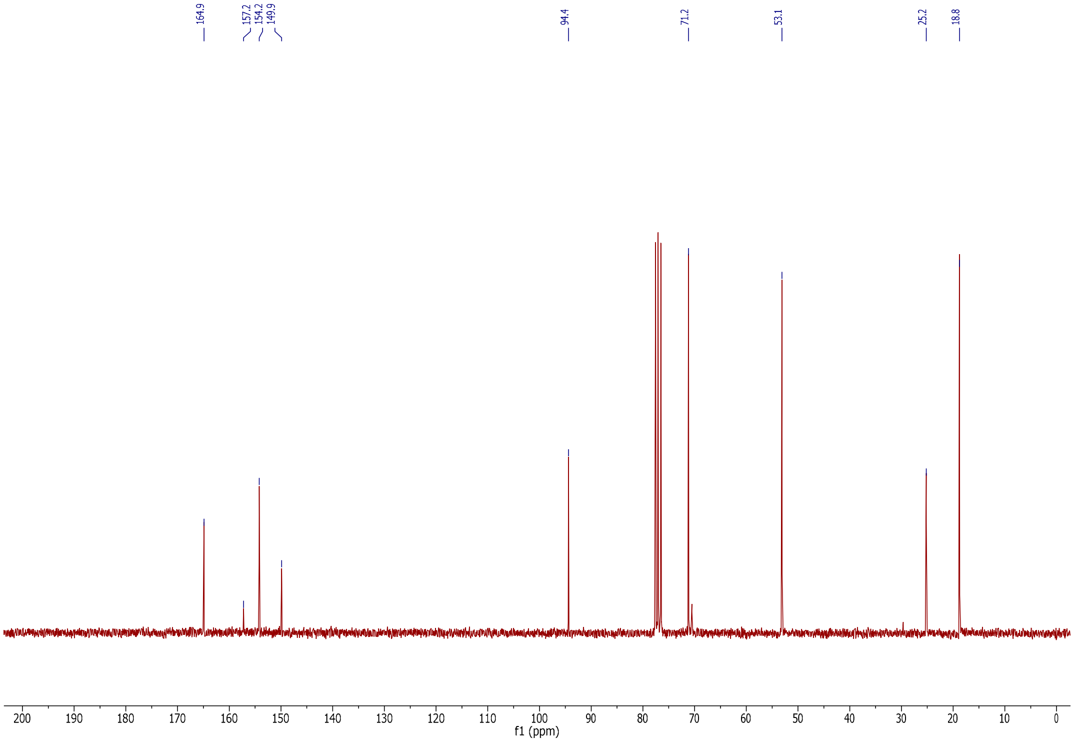
164.9
157.2
154.2
149.9
94.4
71.2
53.1
25.2
18.8
f1 (ppm)

Compound 10

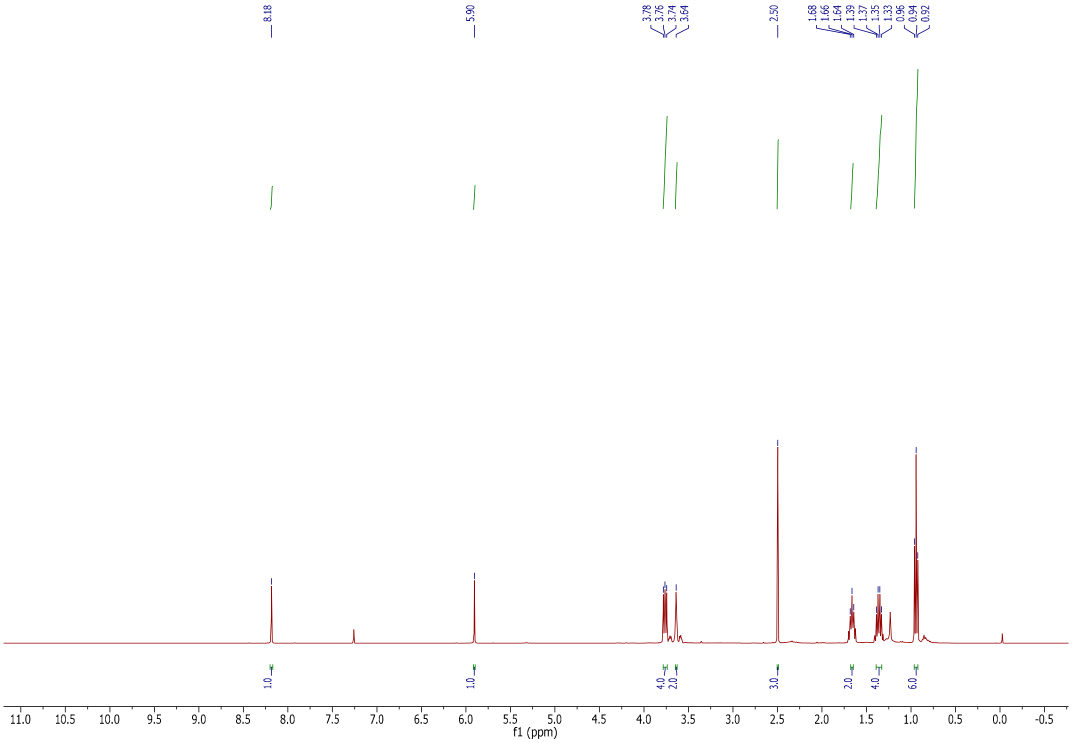
8.18
5.90
3.78
3.76
3.74
3.64
2.50
1.68
1.66
1.64
1.39
1.37
1.35
1.33
0.96
0.94
0.92
1.0
1.0
4.0
2.0
3.0
2.0
4.0
6.0
f1 (ppm)

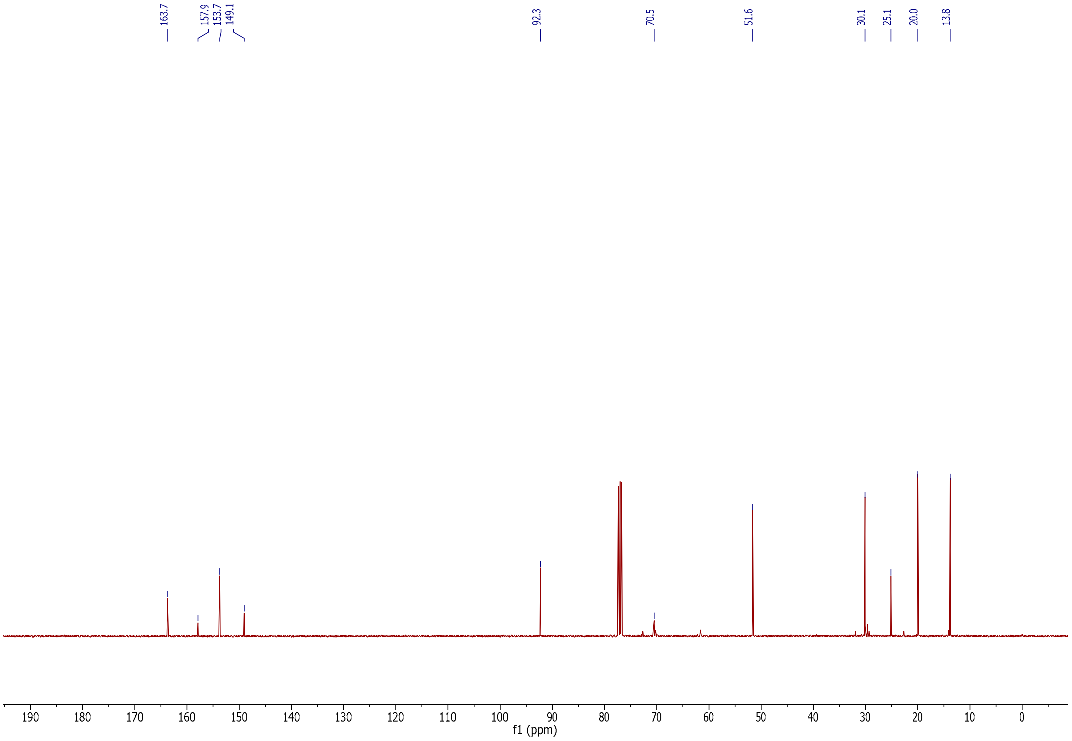
163.7
157.9
153.7
149.1
92.3
70.5
51.6
30.1
25.1
20.0
13.8
f1 (ppm)

Compound 11

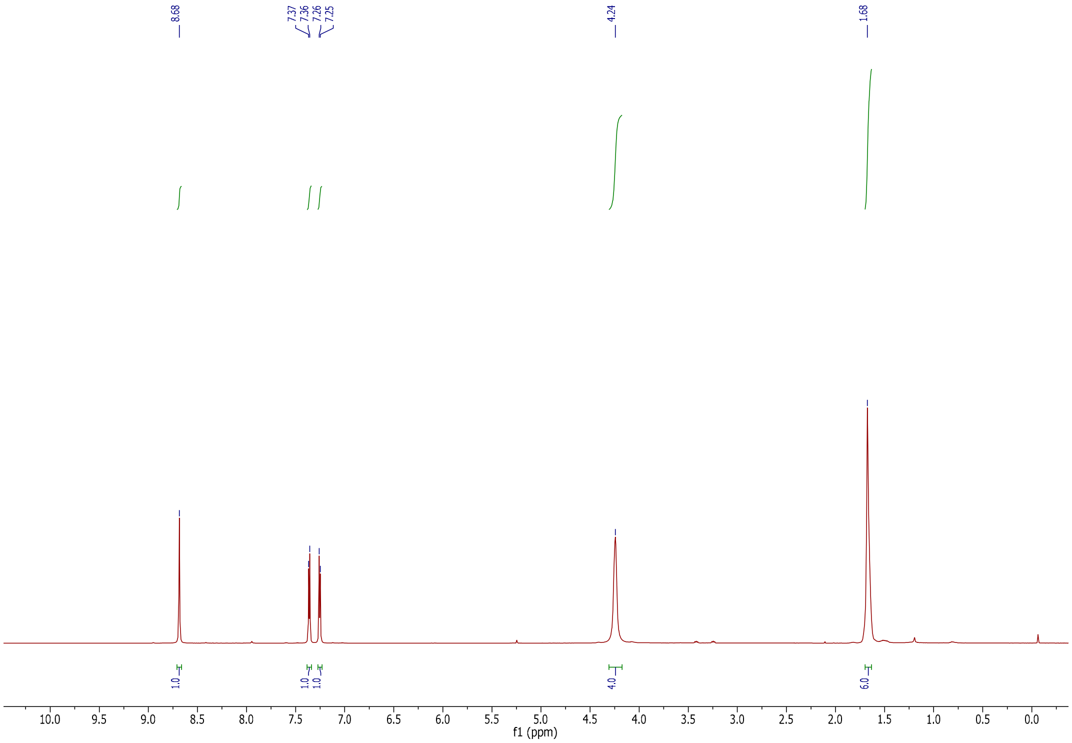
8.68
7.37
7.36
7.26
7.25
4.24
1.68
1.0
1.0
1.0
4.0
6.0
10.0 9.5 9.0 8.5 8.0 7.5 7.0 6.5 6.0 5.5 5.0 4.5 4.0 3.5 3.0 2.5 2.0 1.5 1.0 0.5 0.0
f1 (ppm)

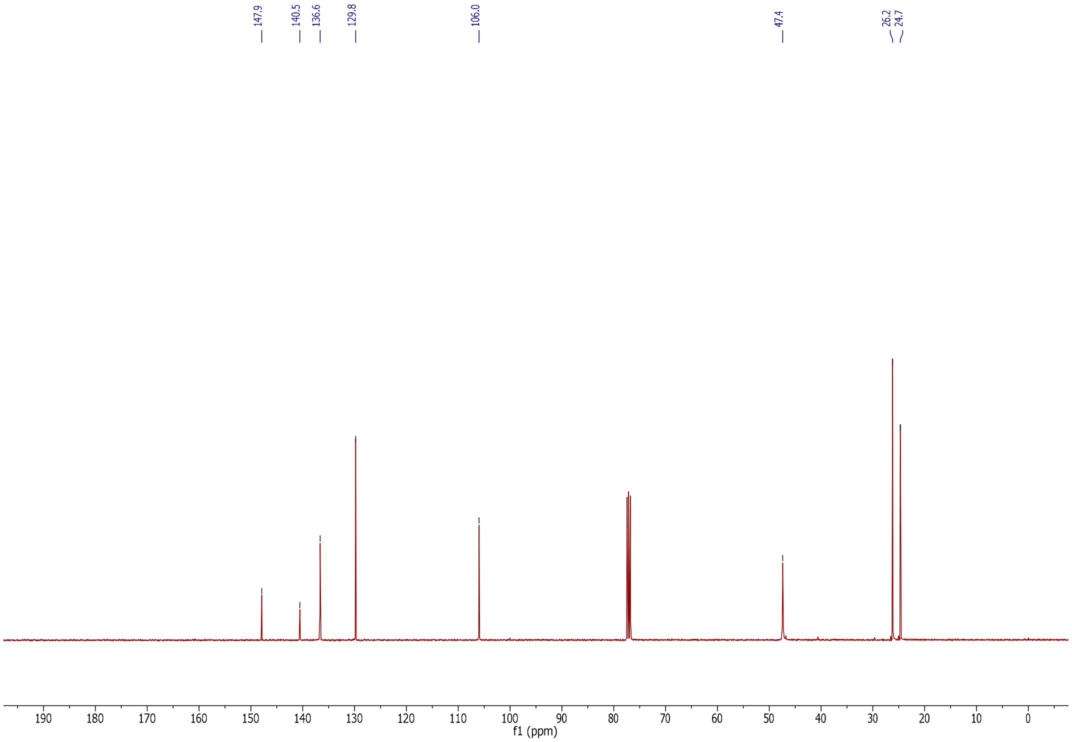
147.9
140.5
136.6
129.8
106.0
47.4
26.2
24.7
190 180 170 160 150 140 130 120 110 100 90 80 70 60 50 40 30 20 10 0
f1 (ppm)

Compound 12

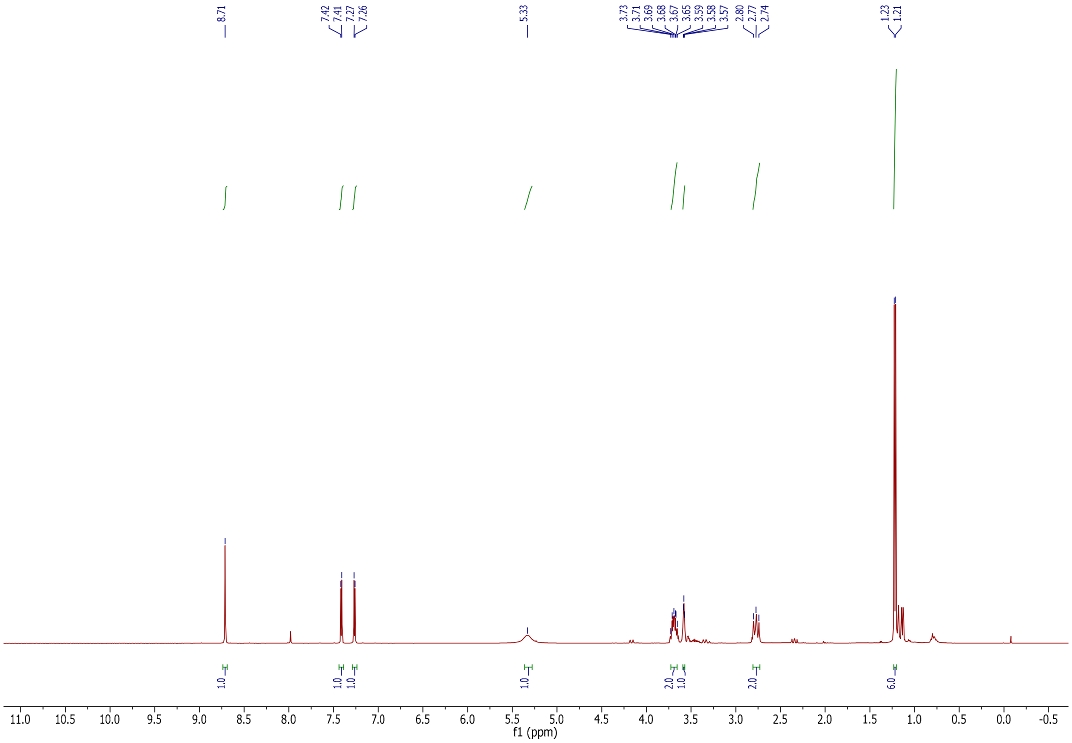

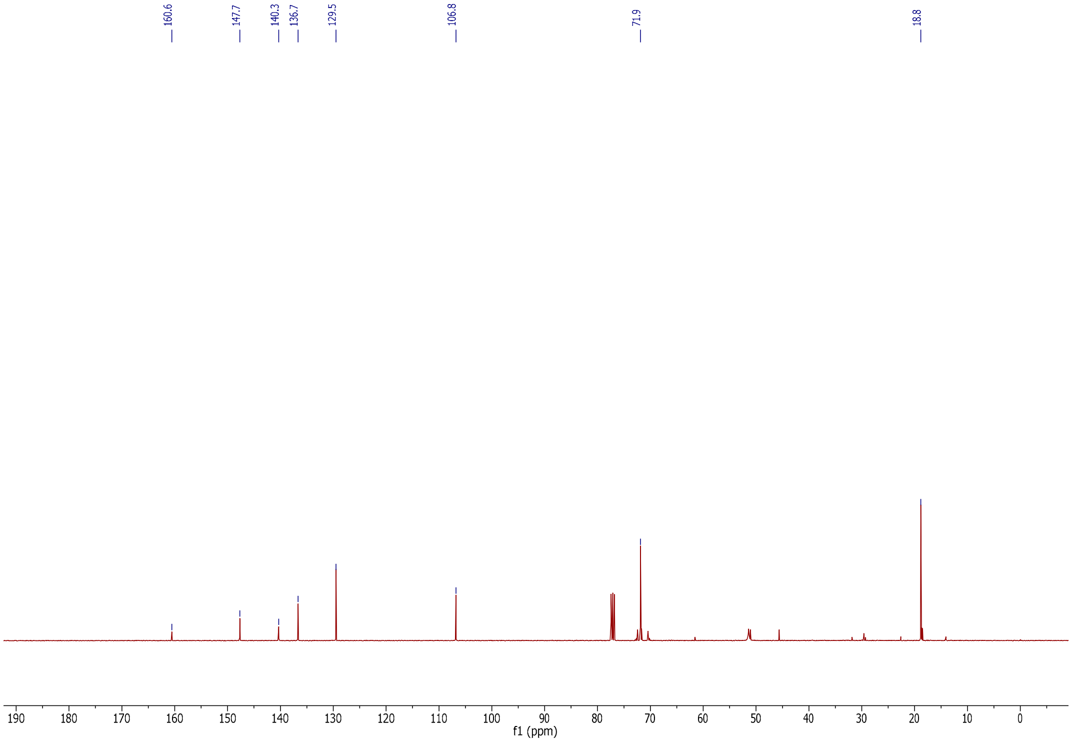

Compound 13

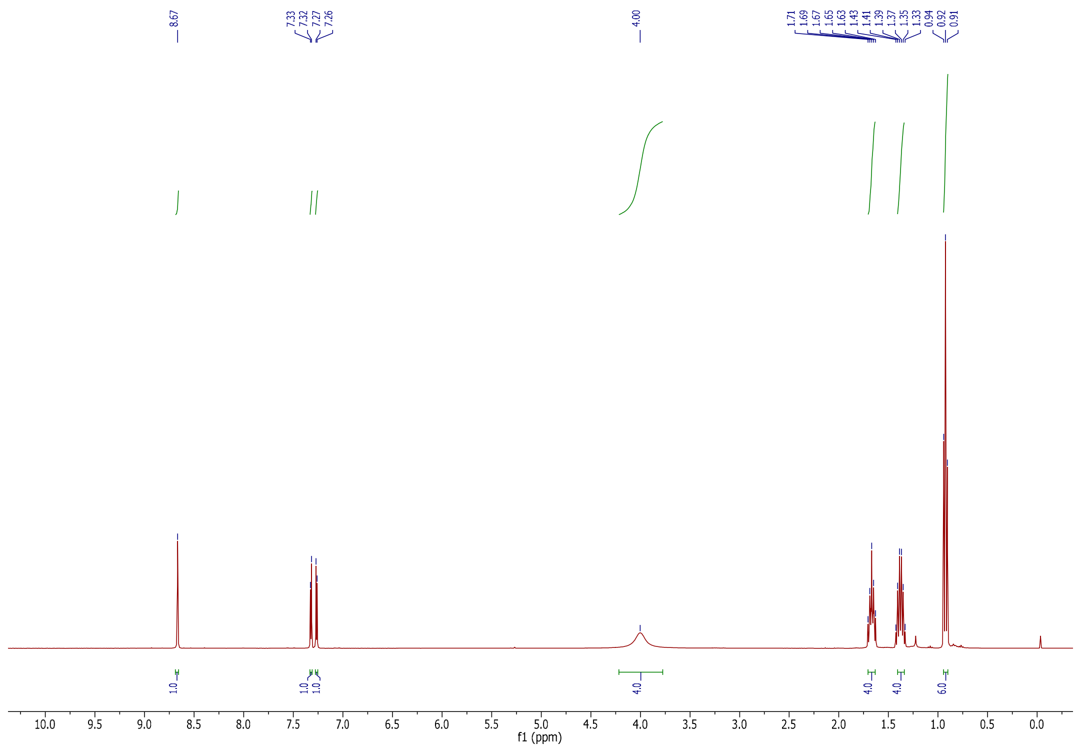
8.67
7.33
7.32
7.27
7.26
4.00
1.71
1.69
1.67
1.65
1.63
1.43
1.41
1.39
1.37
1.35
1.33
0.94
0.92
0.91
1.0
1.0
1.0
4.0
4.0
4.0
6.0
f1 (ppm)

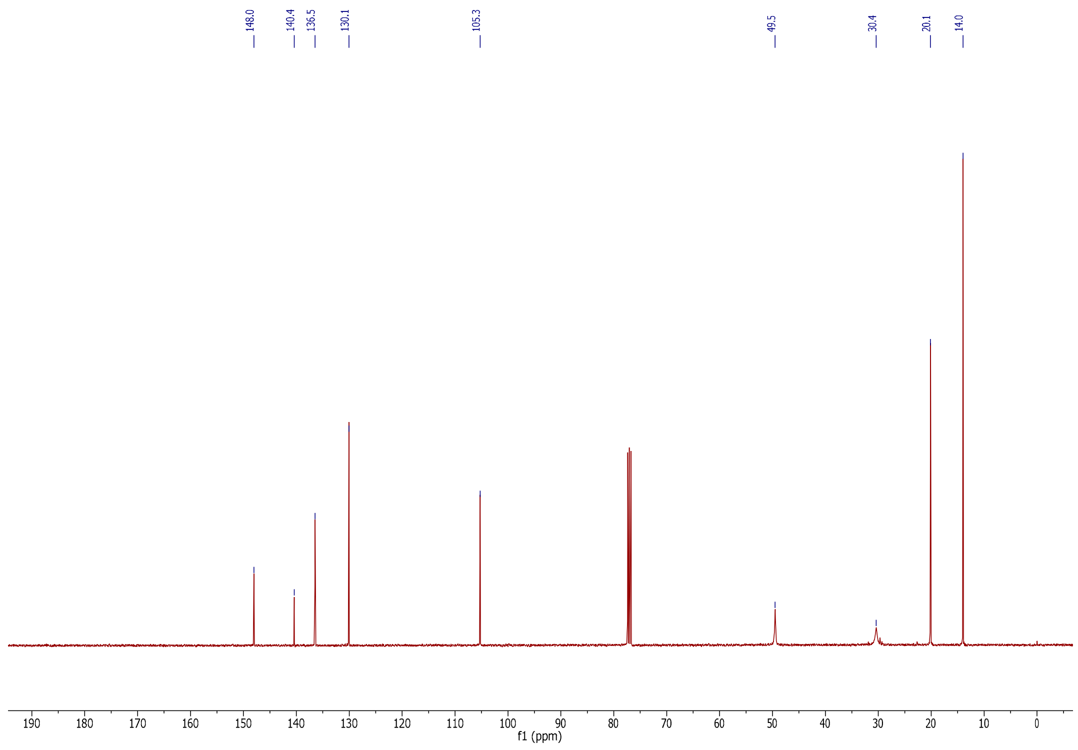
148.0
140.4
136.5
130.1
105.3
49.5
30.4
20.1
14.0
f1 (ppm)

Compound 14

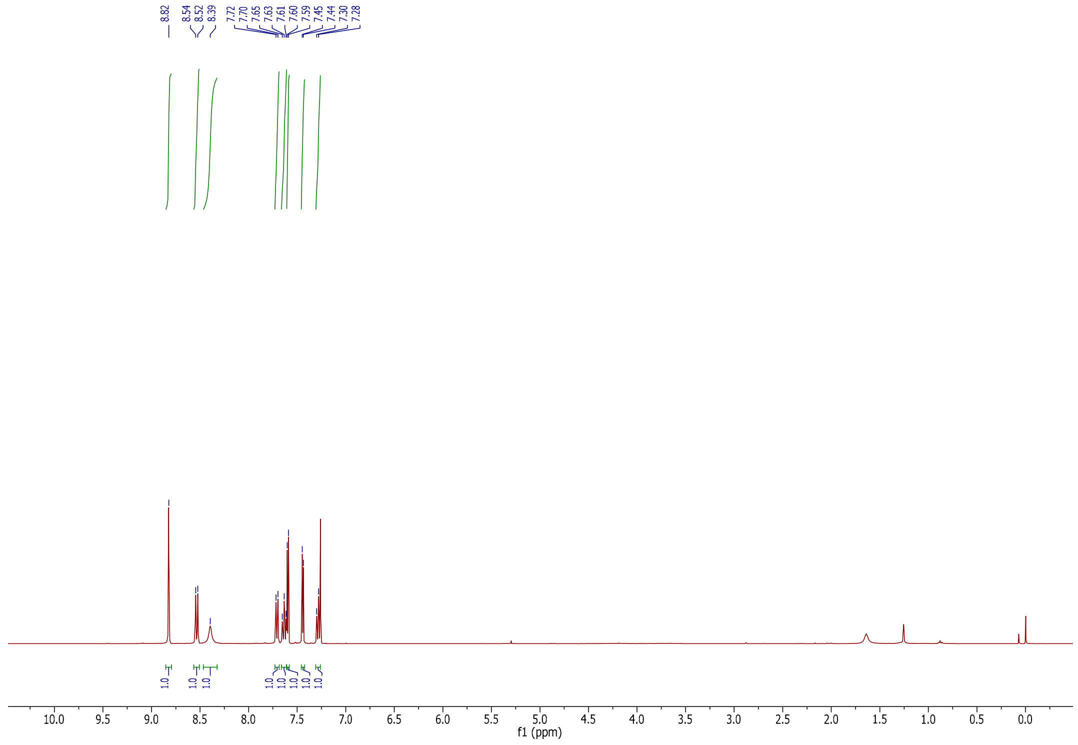
8.82
8.54
8.52
8.39
7.72
7.70
7.65
7.63
7.61
7.60
7.59
7.45
7.44
7.30
7.28
1.0
1.0
1.0
1.0
1.0
1.0
1.0
1.0
10.0
9.5
9.0
8.5
8.0
7.5
7.0
6.5
6.0
5.5
5.0
4.5
4.0
3.5
3.0
2.5
2.0
1.5
1.0
0.5
0.0
f1 (ppm)

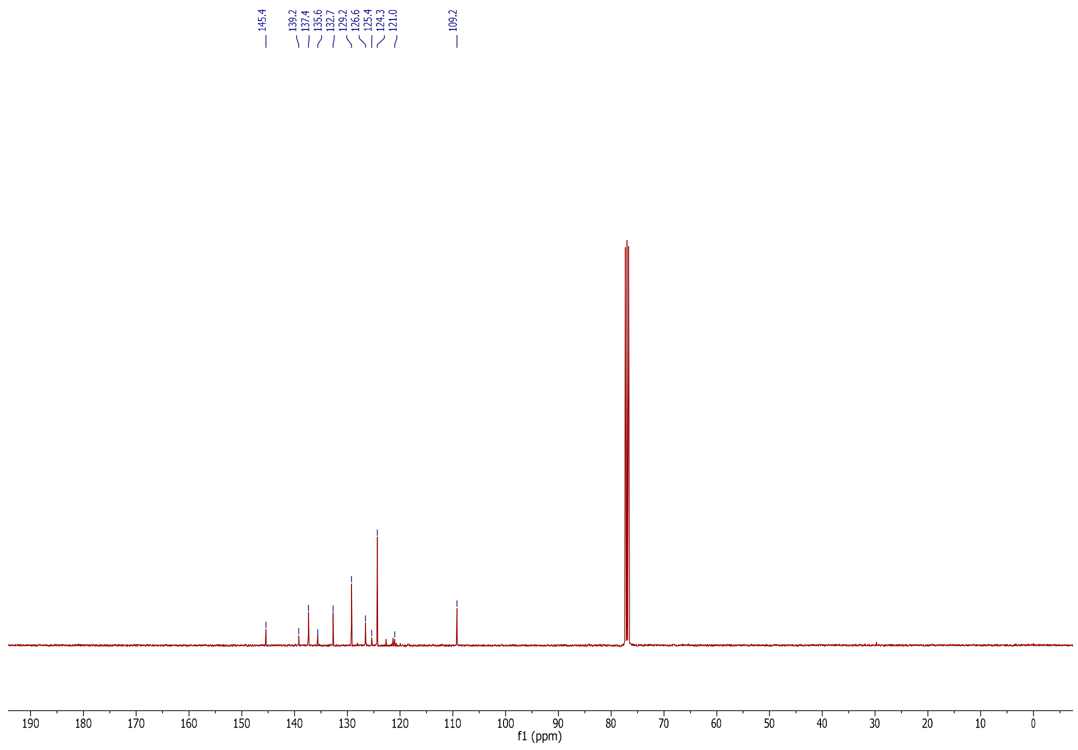
145.4
139.2
137.4
135.6
132.7
129.2
126.6
125.4
124.3
121.0
109.2
190
180
170
160
150
140
130
120
110
100
90
80
70
60
50
40
30
20
10
0
f1 (ppm)

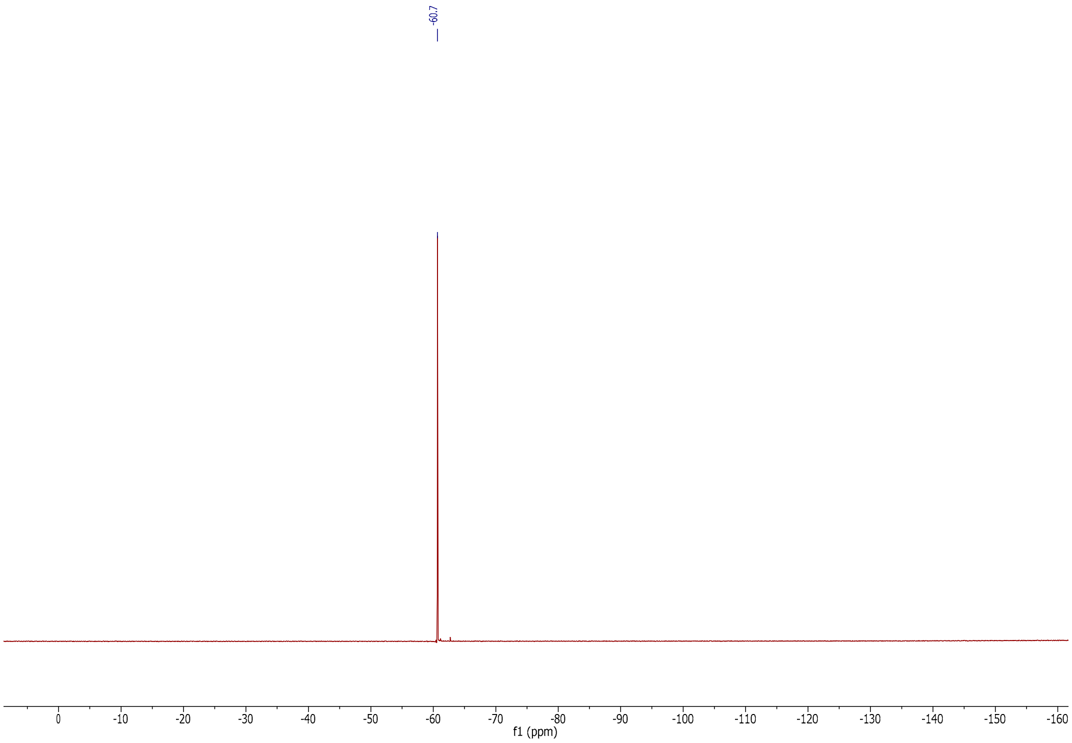

-60.7
0
-10
-20
-30
-40
-50
-60
-70
-80
-90
-100
-110
-120
-130
-140
-150
-160
f1 (ppm)

Compound 15

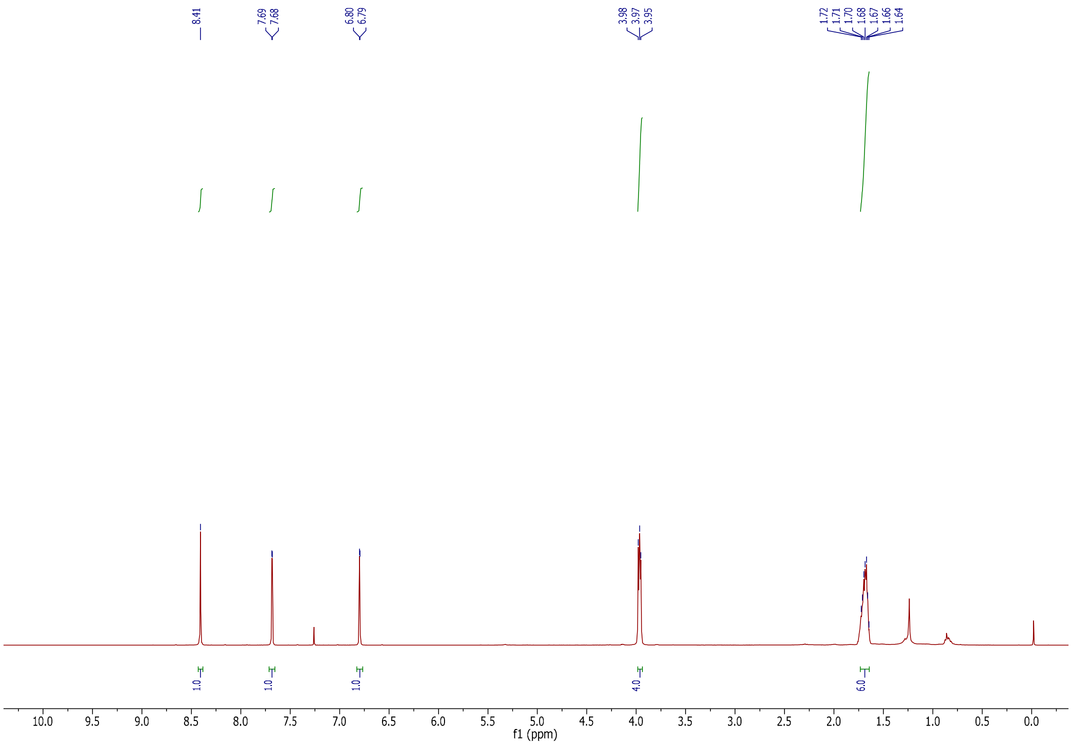

8.41
7.69
7.68
6.80
6.79
3.98
3.97
3.95
1.72
1.71
1.70
1.68
1.67
1.66
1.64
1.0
1.0
1.0
4.0
6.0
10.0
9.5
9.0
8.5
8.0
7.5
7.0
6.5
6.0
5.5
5.0
4.5
4.0
3.5
3.0
2.5
2.0
1.5
1.0
0.5
0.0
f1 (ppm)

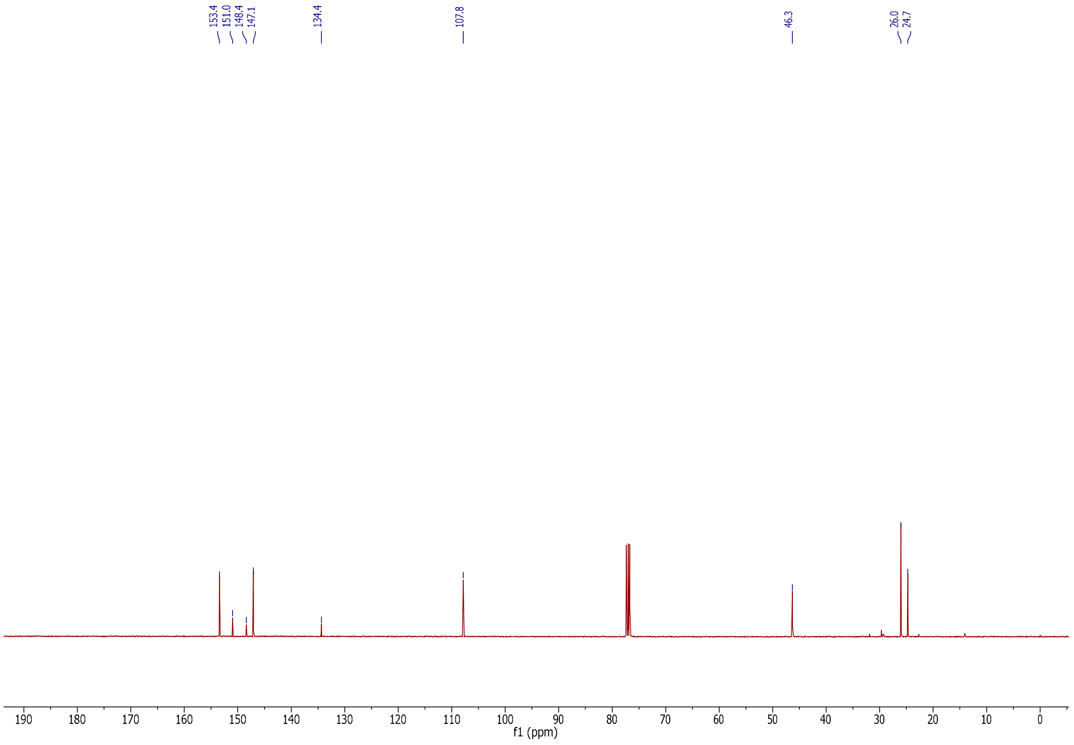
153.4
151.0
148.4
147.1
134.4
107.8
46.3
26.0
24.7
f1 (ppm)

Compound 16

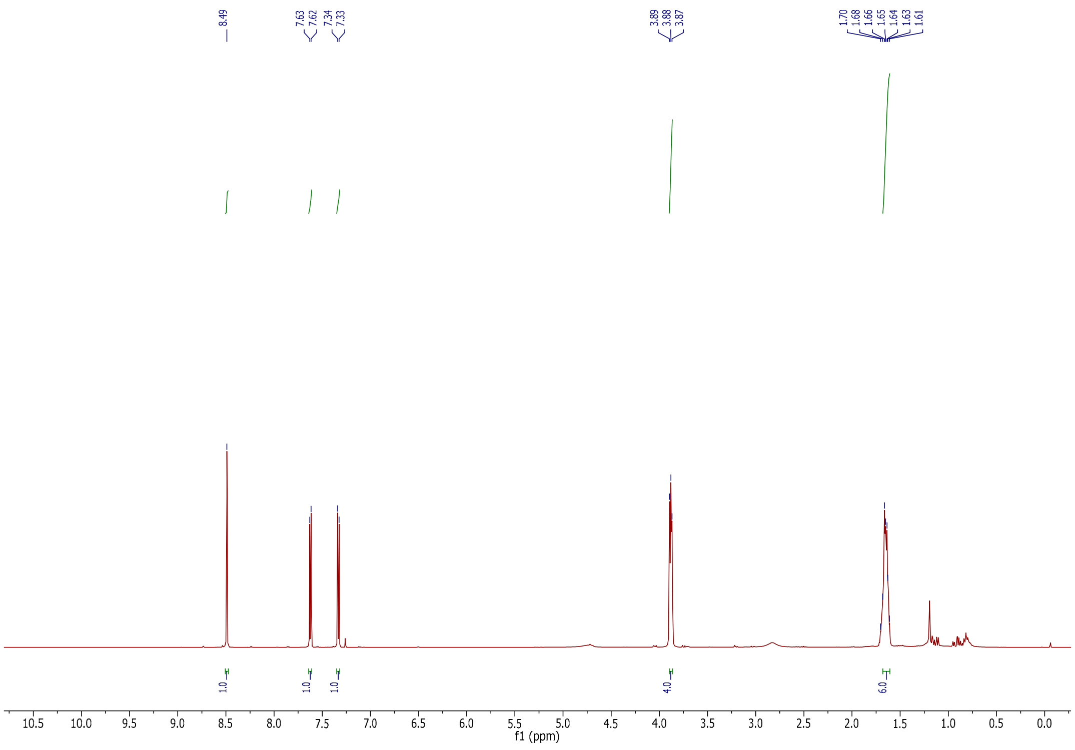
8.49
7.63
7.62
7.34
7.33
3.89
3.88
3.87
1.70
1.68
1.66
1.65
1.64
1.63
1.61
1.0
1.0
1.0
4.0
6.0
f1 (ppm)

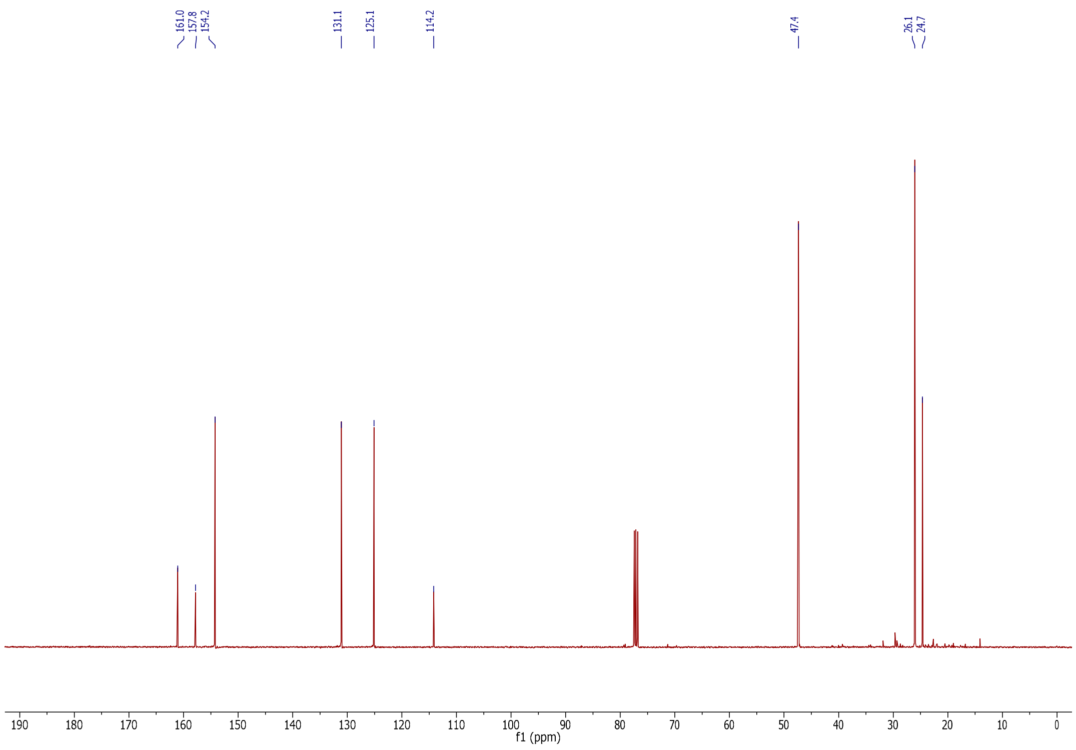
161.0
157.8
154.2
131.1
125.1
114.2
47.4
26.1
24.7
190 180 170 160 150 140 130 120 110 100 90 80 70 60 50 40 30 20 10 0
f1 (ppm)

Compound 17

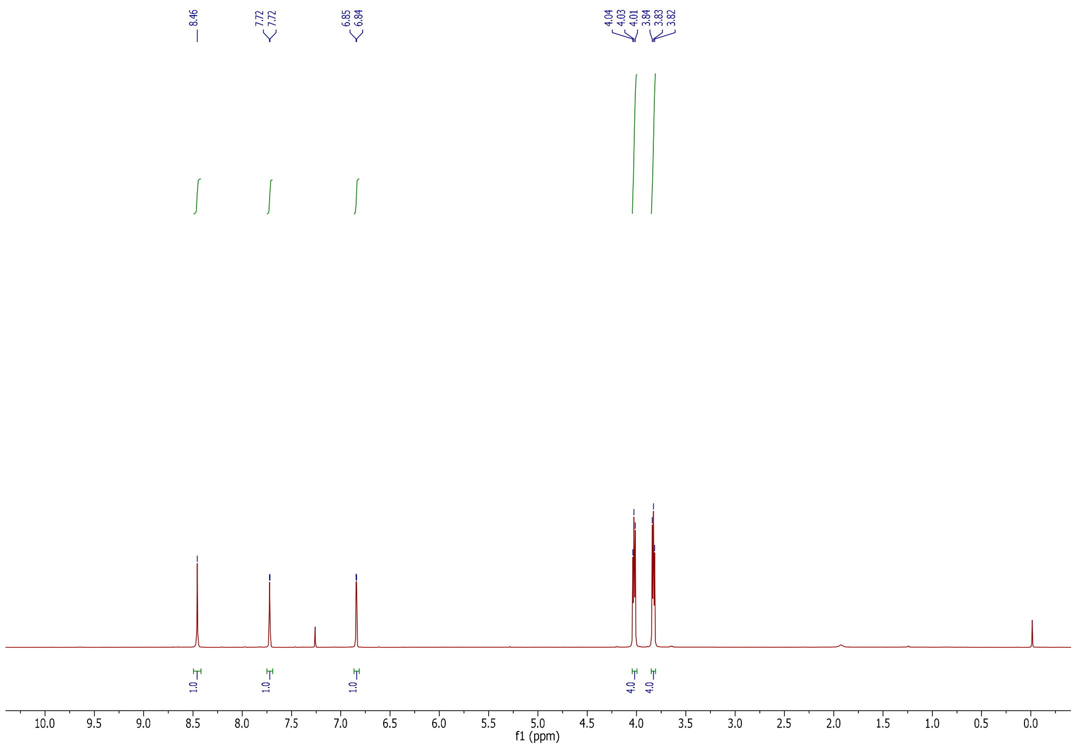
8.46
7.72
7.72
6.85
6.84
4.04
4.03
4.01
3.84
3.83
3.82
1.0
1.0
1.0
4.0
4.0
10.0 9.5 9.0 8.5 8.0 7.5 7.0 6.5 6.0 5.5 5.0 4.5 4.0 3.5 3.0 2.5 2.0 1.5 1.0 0.5 0.0
f1 (ppm)

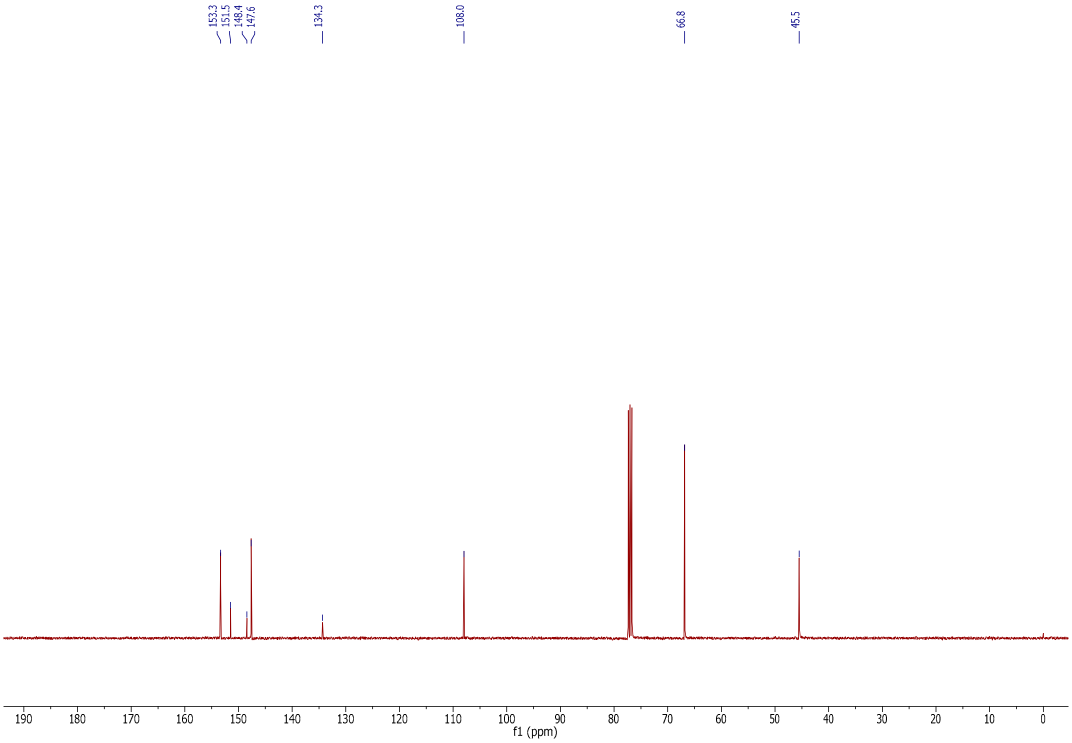
153.3
151.5
148.4
147.6
134.3
108.0
66.8
45.5
190 180 170 160 150 140 130 120 110 100 90 80 70 60 50 40 30 20 10 0
f1 (ppm)

Compound 18

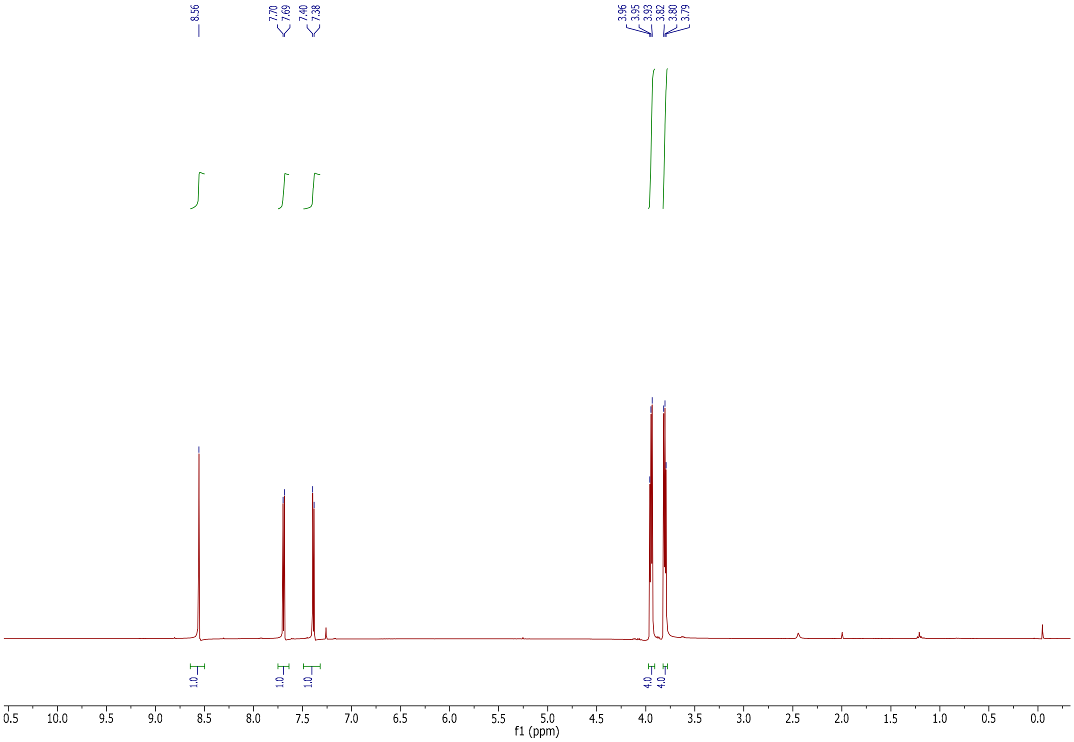
8.56
7.70
7.69
7.40
7.38
3.96
3.95
3.93
3.82
3.80
3.79
1.0
1.0
1.0
4.0
4.0
0.5 10.0 9.5 9.0 8.5 8.0 7.5 7.0 6.5 6.0 5.5 5.0 4.5 4.0 3.5 3.0 2.5 2.0 1.5 1.0 0.5 0.0
f1 (ppm)

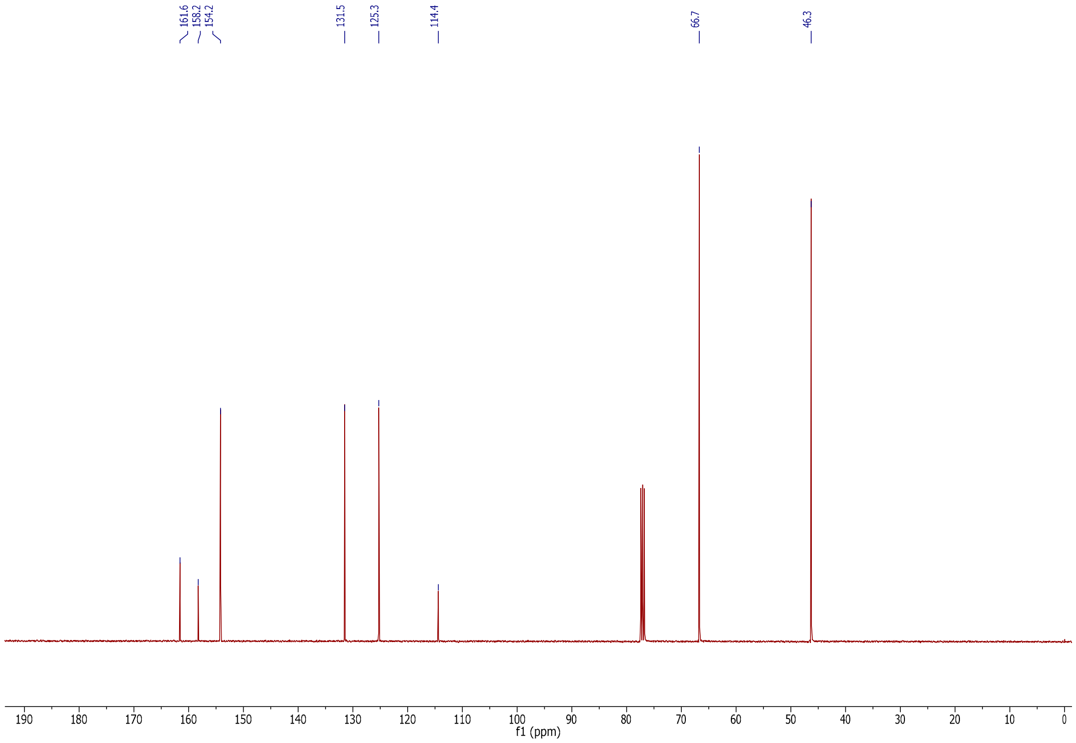
161.6
158.2
154.2
131.5
125.3
114.4
66.7
46.3
190 180 170 160 150 140 130 120 110 100 90 80 70 60 50 40 30 20 10 0
f1 (ppm)

Compound 19

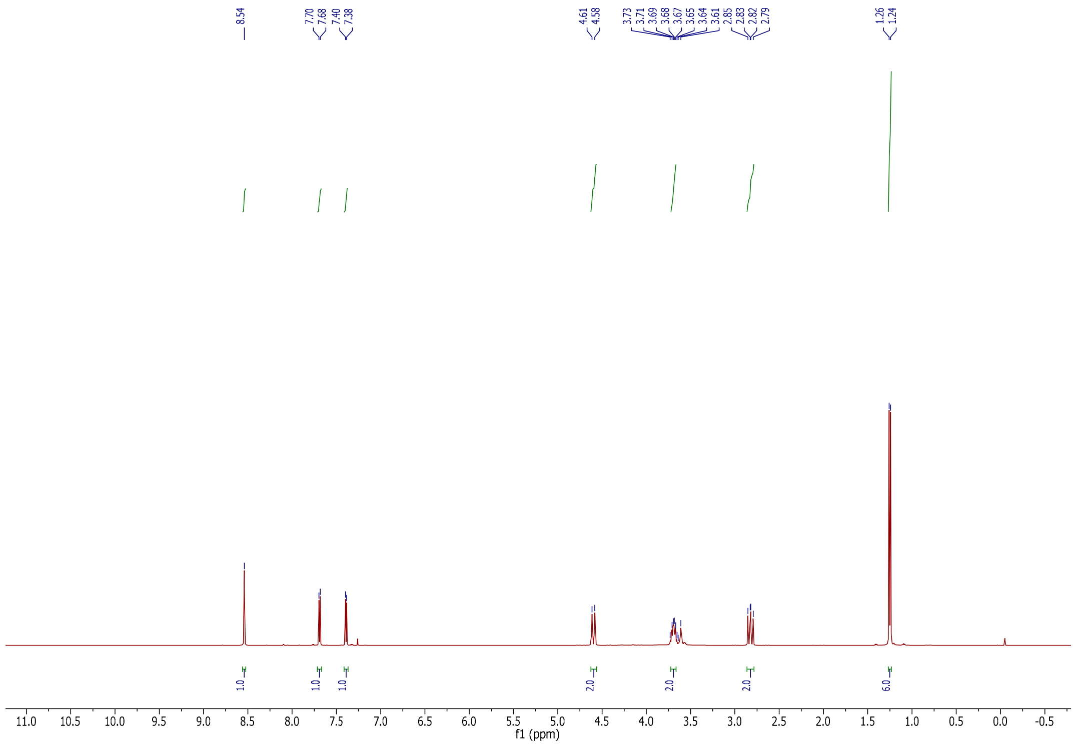
8.54
7.70
7.68
7.40
7.38
4.61
4.58
3.73
3.71
3.69
3.68
3.67
3.65
3.64
3.61
2.85
2.83
2.82
2.79
1.26
1.24
1.0
1.0
1.0
2.0
2.0
2.0
6.0
11.0 10.5 10.0 9.5 9.0 8.5 8.0 7.5 7.0 6.5 6.0 5.5 5.0 4.5 4.0 3.5 3.0 2.5 2.0 1.5 1.0 0.5 0.0 -0.5
f1 (ppm)

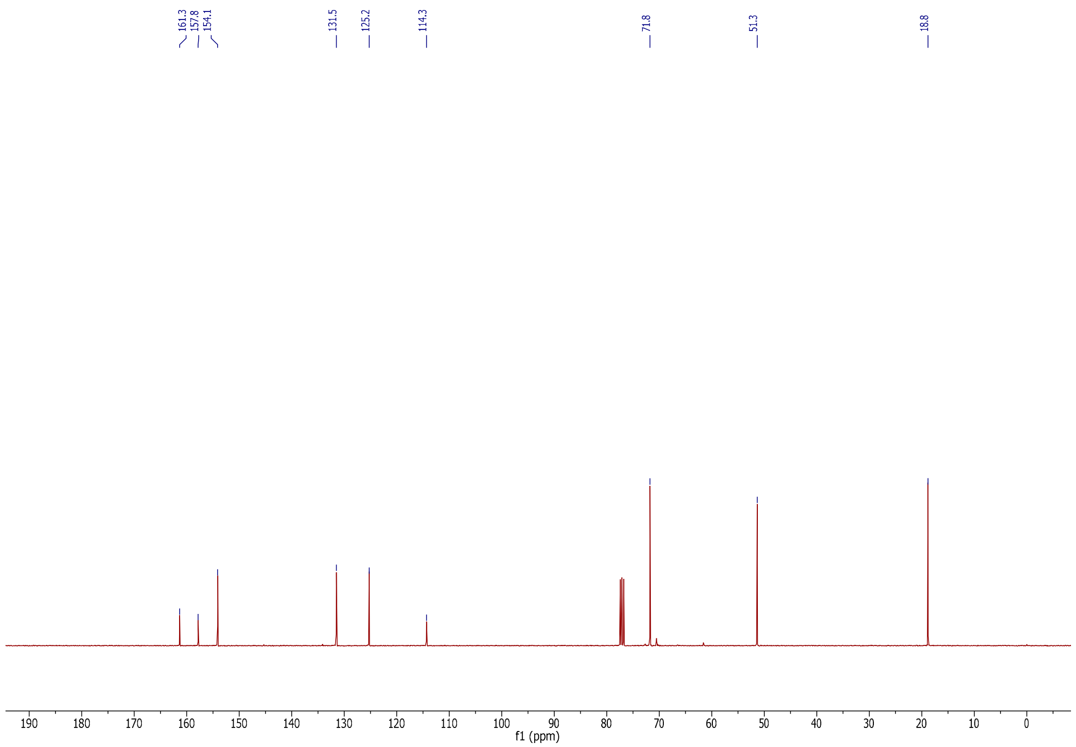
161.3
157.8
154.1
131.5
125.2
114.3
71.8
51.3
18.8
f1 (ppm)

Compound 20

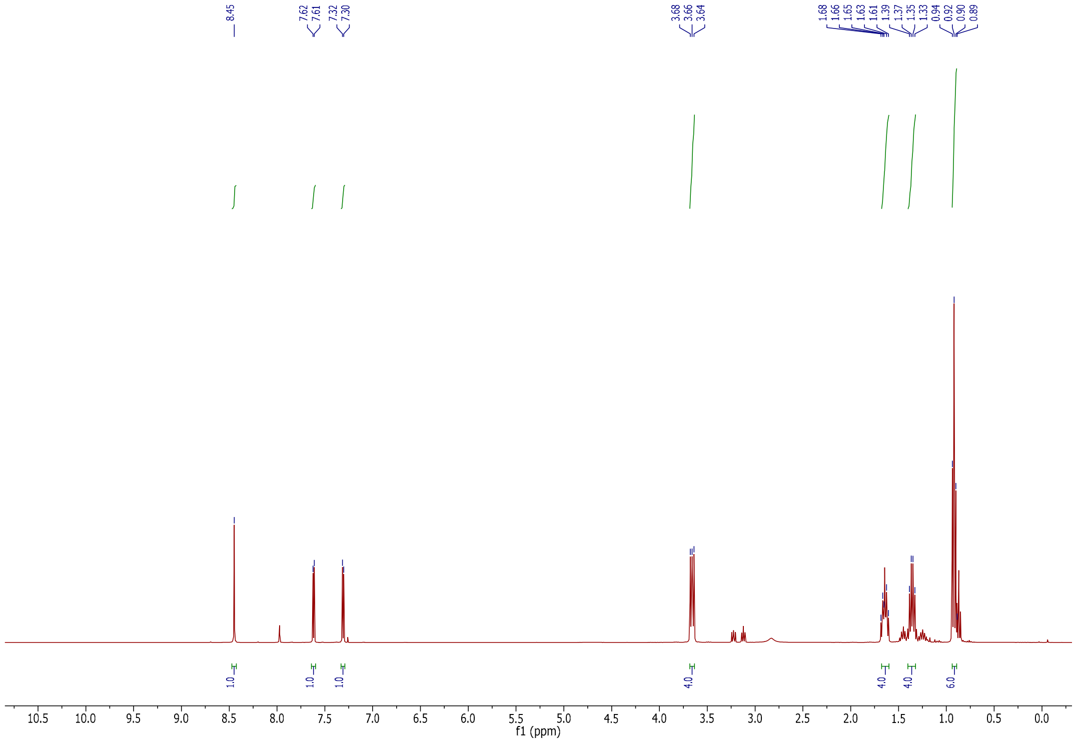
8.45
7.62
7.61
7.32
7.30
3.68
3.66
3.64
1.68
1.66
1.65
1.63
1.61
1.39
1.37
1.35
1.33
0.94
0.92
0.90
0.89
f1 (ppm)

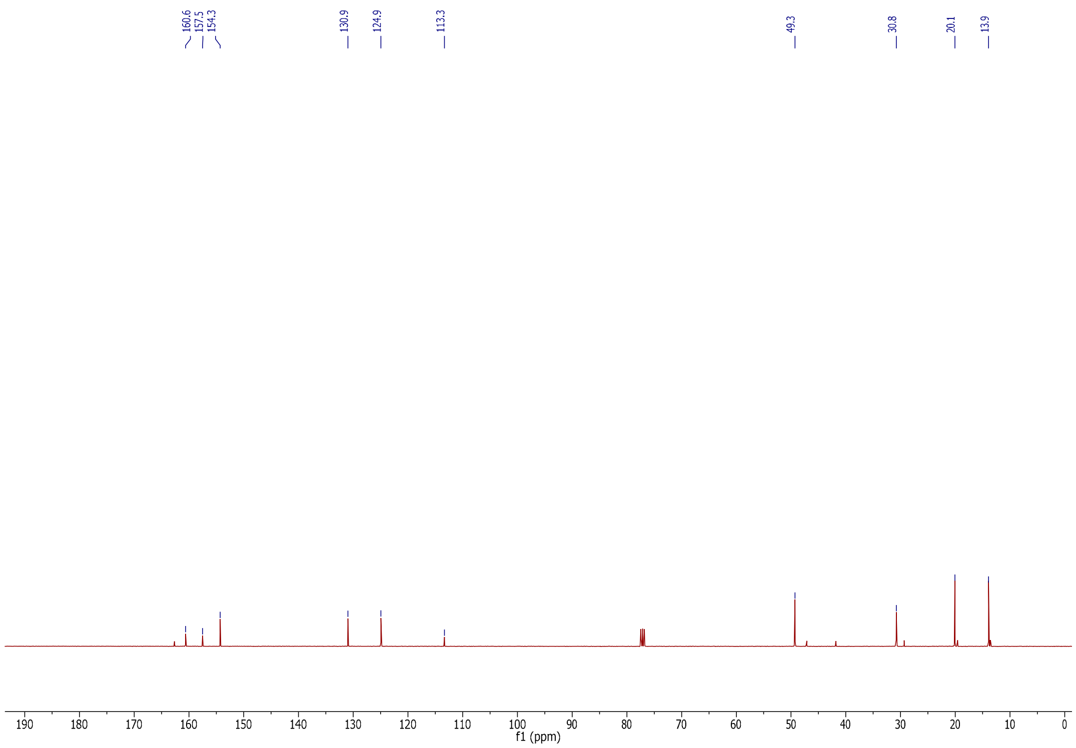
160.6
157.5
154.3
130.9
124.9
113.3
49.3
30.8
20.1
13.9
190 180 170 160 150 140 130 120 110 100 90 80 70 60 50 40 30 20 10 0
f1 (ppm)

Compound 21

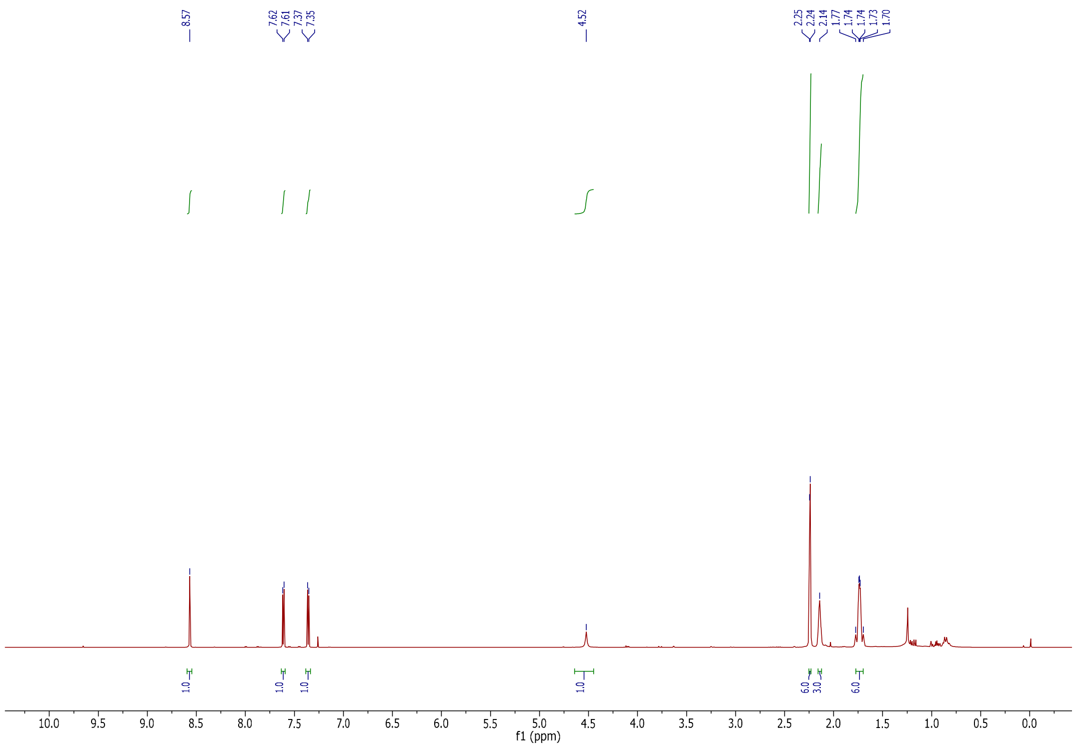
8.57
7.62
7.61
7.37
7.35
4.52
2.25
2.24
2.14
1.77
1.74
1.74
1.73
1.70
1.0
1.0
1.0
1.0
6.0
3.0
6.0
10.0 9.5 9.0 8.5 8.0 7.5 7.0 6.5 6.0 5.5 5.0 4.5 4.0 3.5 3.0 2.5 2.0 1.5 1.0 0.5 0.0
f1 (ppm)

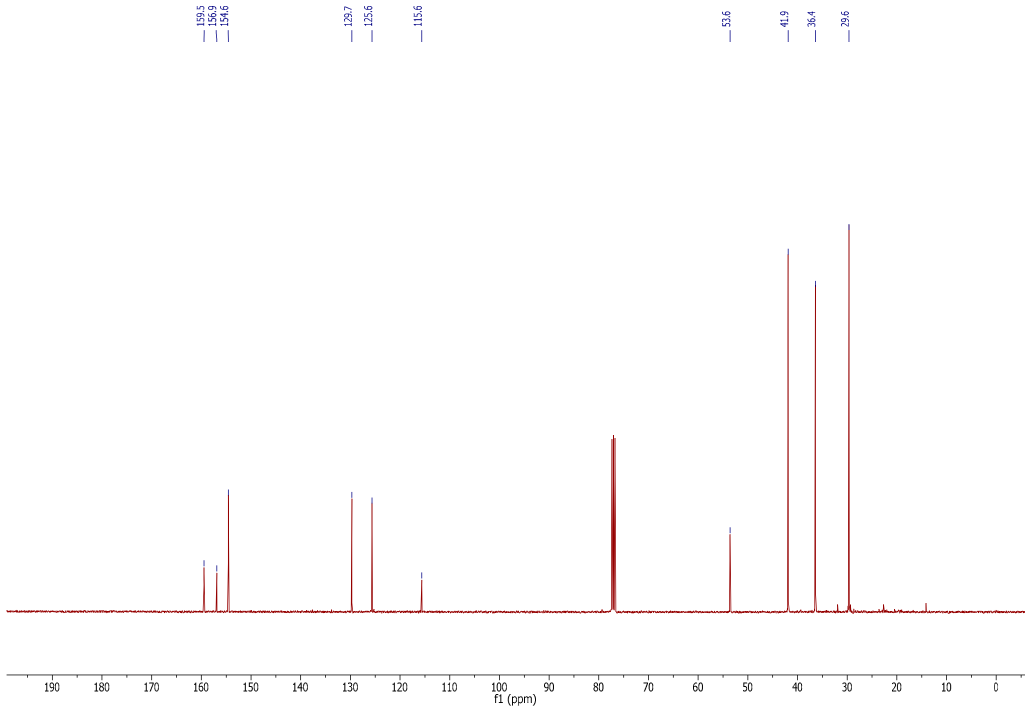
159.5
156.9
154.6
129.7
125.6
115.6
53.6
41.9
36.4
29.6
f1 (ppm)

Compound 22

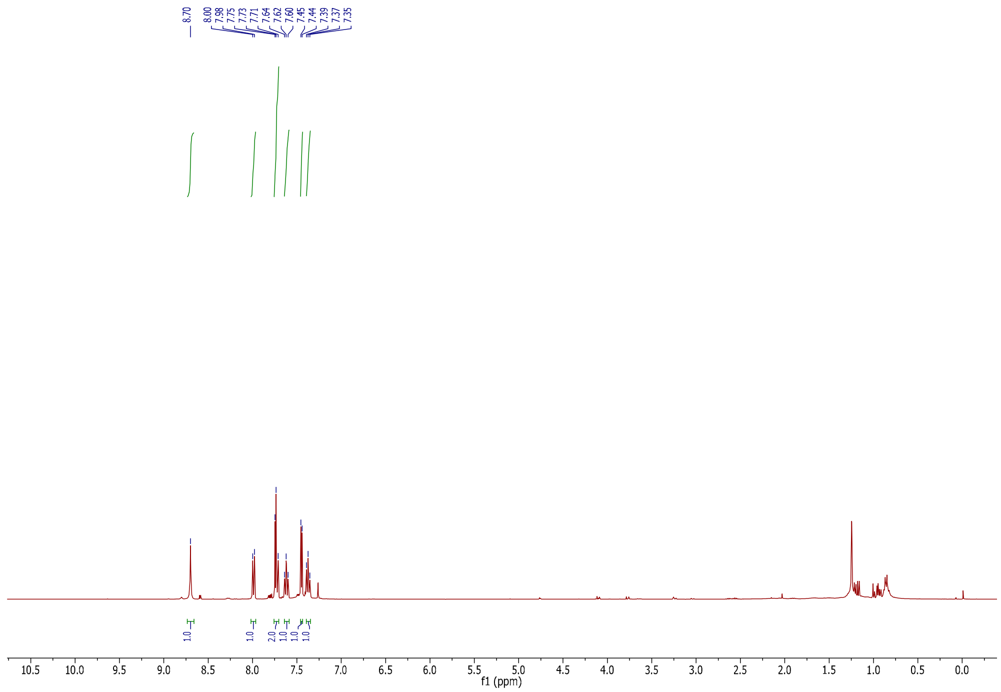
8.70
8.00
7.98
7.75
7.73
7.71
7.64
7.62
7.60
7.45
7.44
7.39
7.37
7.35
f1 (ppm)

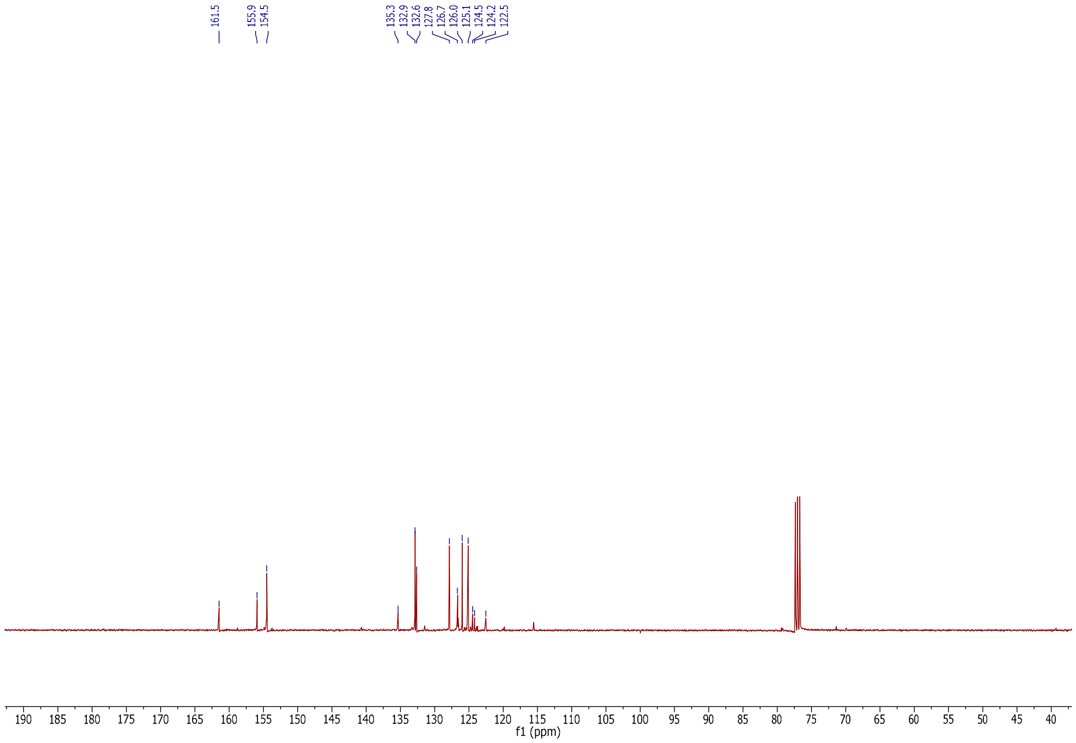
161.5
155.9
154.5
135.3
132.9
132.6
127.8
126.7
126.0
125.1
124.5
124.2
122.5
f1 (ppm)

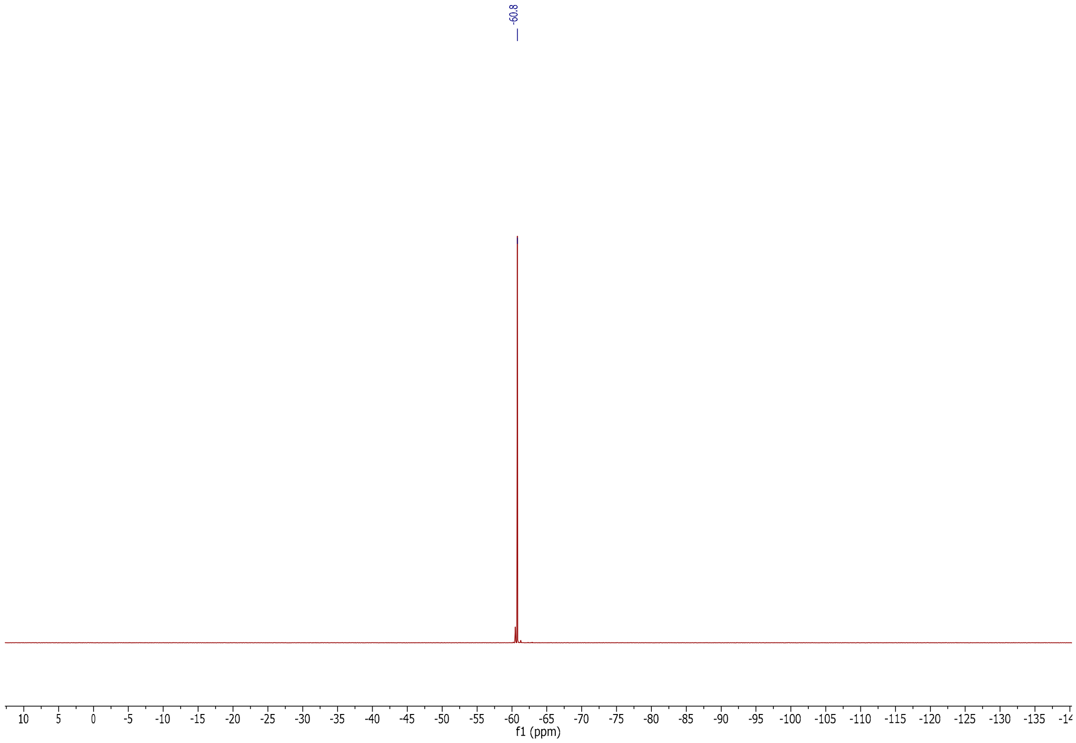
-60.8
f1 (ppm)

Compound 23

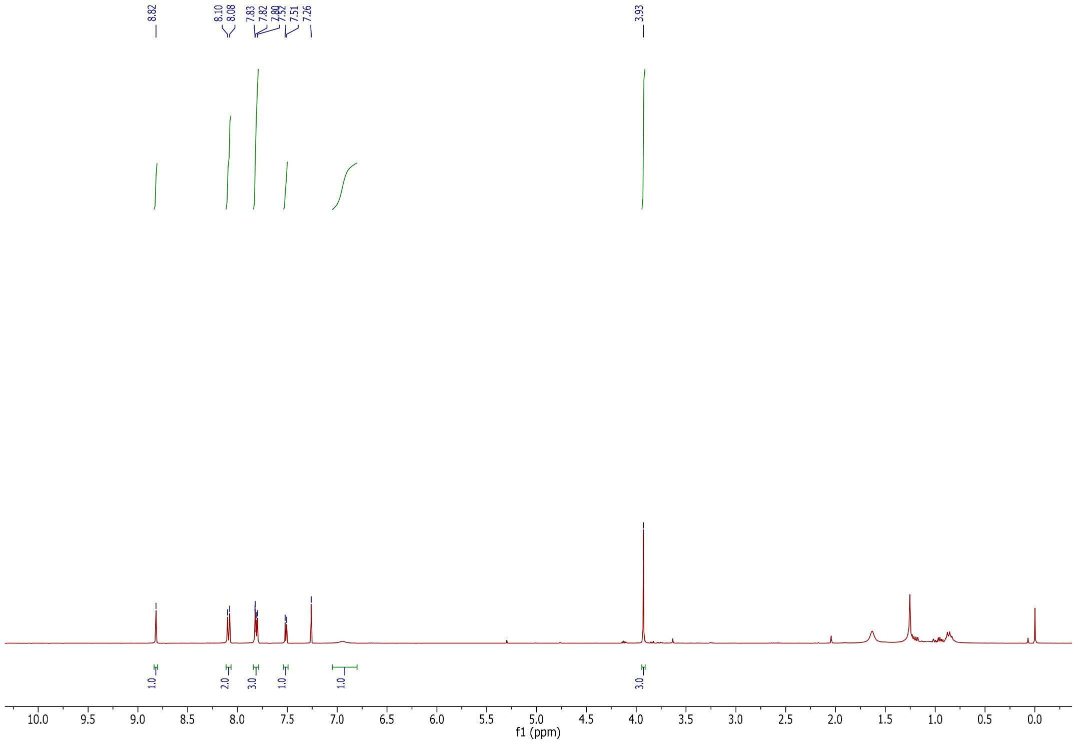
8.82
8.10
8.08
7.83
7.82
7.80
7.52
7.51
7.26
3.93
1.0
2.0
3.0
1.0
1.0
3.0
10.0 9.5 9.0 8.5 8.0 7.5 7.0 6.5 6.0 5.5 5.0 4.5 4.0 3.5 3.0 2.5 2.0 1.5 1.0 0.5 0.0
f1 (ppm)

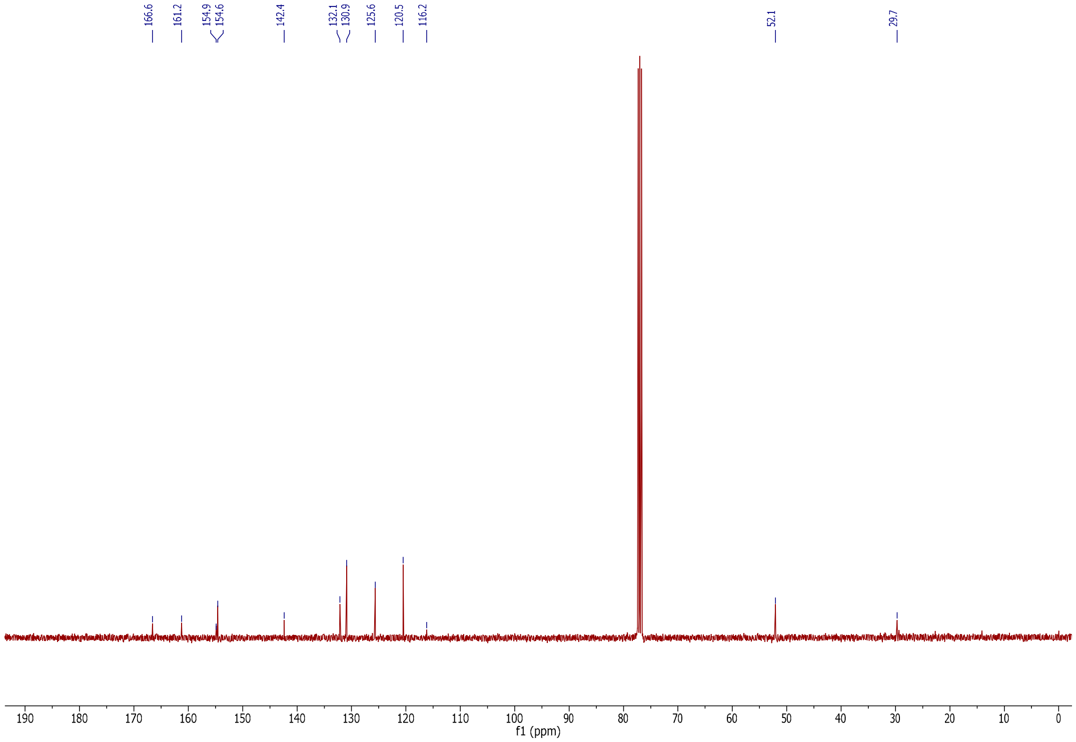
166.6
161.2
154.9
154.6
142.4
132.1
130.9
125.6
120.5
116.2
52.1
29.7
190 180 170 160 150 140 130 120 110 100 90 80 70 60 50 40 30 20 10 0
f1 (ppm)

Compound 24

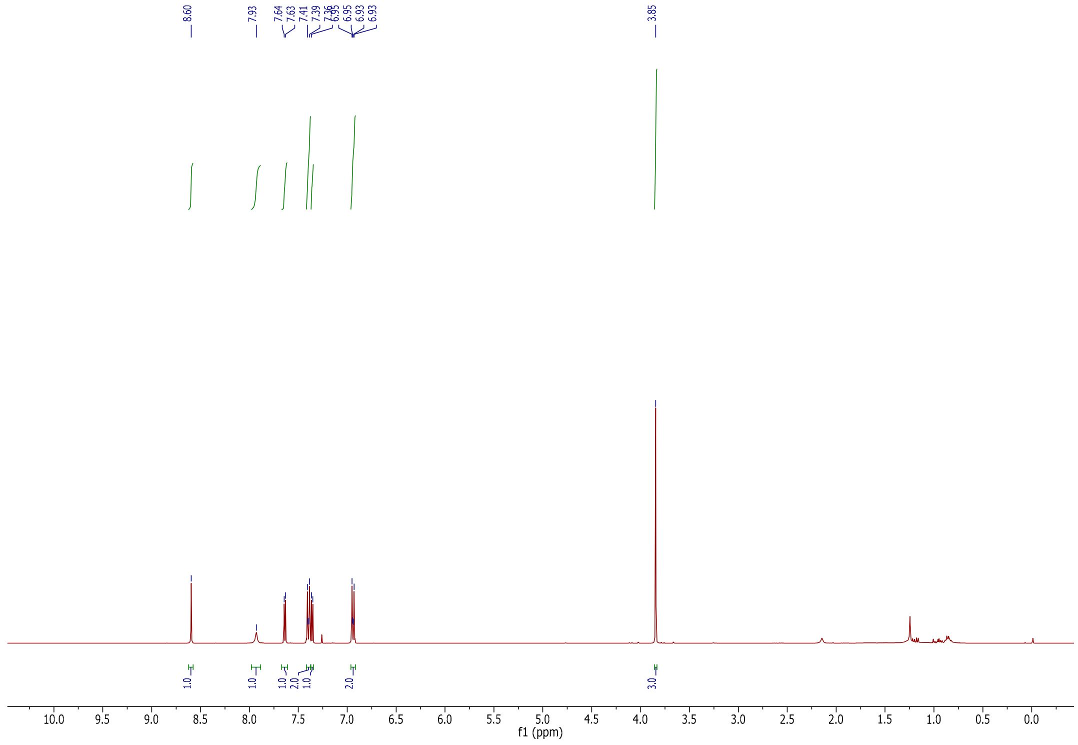
8.60
7.93
7.64
7.63
7.41
7.39
7.36
6.95
6.95
6.93
6.93
3.85
1.0
1.0
1.0
2.0
1.0
2.0
3.0
10.0 9.5 9.0 8.5 8.0 7.5 7.0 6.5 6.0 5.5 5.0 4.5 4.0 3.5 3.0 2.5 2.0 1.5 1.0 0.5 0.0
f1 (ppm)

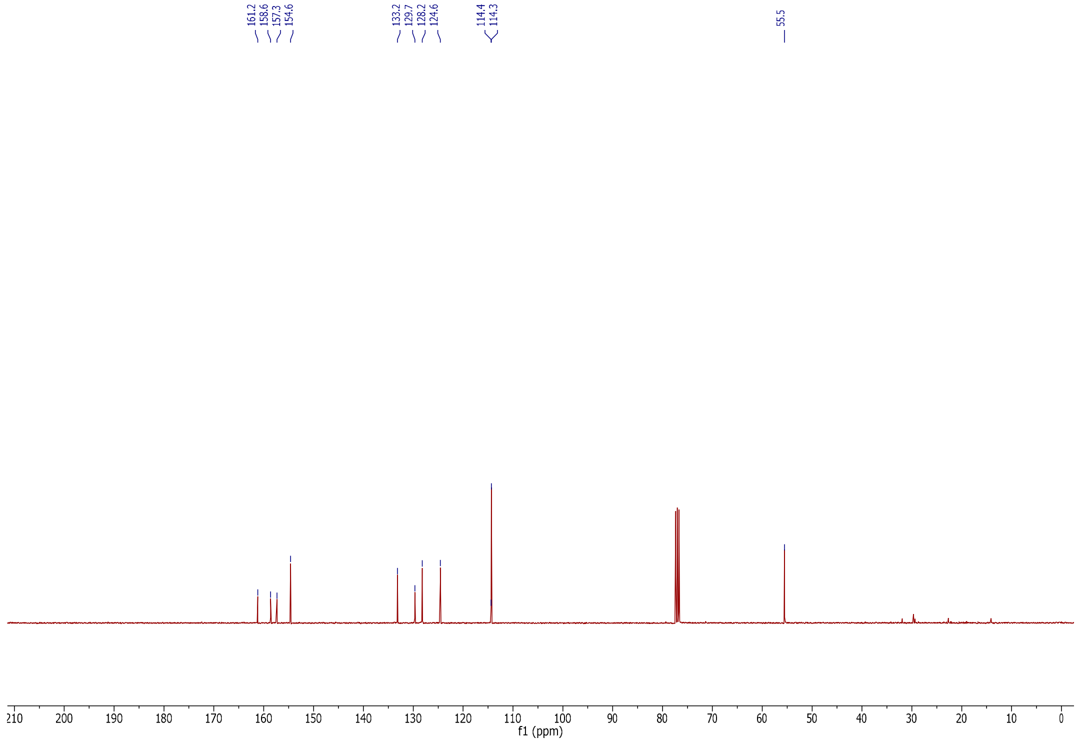
161.2
158.6
157.3
154.6
133.2
129.7
128.2
124.6
114.4
114.3
55.5
210 200 190 180 170 160 150 140 130 120 110 100 90 80 70 60 50 40 30 20 10 0
f1 (ppm)
